# Research the effect of anticipated regret and fairness concerns on retailer-led supply chain

**DOI:** 10.1371/journal.pone.0279334

**Published:** 2023-01-18

**Authors:** Jin Liu, Qi Tian, Nian Zhang

**Affiliations:** 1 School of Economics and Management, Chongqing University of Posts and Telecommunications, Chongqing, P.R. China; 2 School of Modern Posts, Chongqing University of Posts and Telecommunications, Chongqing, P.R. China; 3 Chongqing Municipal Education Commission Digital Economy Innovation and Industrial Development Research Team, Chongqing, P.R. China; Szechenyi Istvan University: Szechenyi Istvan Egyetem, HUNGARY

## Abstract

Considering the consumer’s anticipated regret caused by price discount, the impact of anticipated regret and manufacturers’ fairness concerns on pricing and profits is explored, and a revenue-sharing contract to optimize the profits of supply chain is explored. In centralized decision-making model with manufacturer’s fairness neutrality and retailer-led decentralized decision-making model with the manufacturer’s fairness concerns, numerical simulation and model comparison is used to analyse regret sensitivity coefficient, consumer heterogeneity, the fairness concern coefficient on pricing decisions and profit coordination. Our results reveal that consumer’s anticipated regret has a negative impact on product prices, retailer’s profit and manufacturer’s profit. Manufacturer’s fairness concerns also increase product price and reduce profits of all parties. Retailer-led supply chain can share the revenue to achieve Pareto optimization. When formulating promotional strategies, retailers should consider the characteristics of anticipated regrets of consumers.

## 1. Introduction

With the popularization of network technology and the rapid development of e-commerce, more and more consumers have adopted the online shopping model, the retailers obtain more comprehensive market information through the direct contact with consumers. The retailers’ advantages have been obvious, so retailers have gradually become the leaders of the supply chain, such as JD and Suning. In addition, the product market has become abundant, and the consumption power of consumers has also been continuously improved, that means consumers are pursuing quality of life. The competition among businesses has become more intense. Both retailer and manufacturer have to adopt different measures to seek more benefits. One of the most common competitive strategies is price discount. From the perspective of a consumer, when faced with a price discount, consumers would be moved by the low price of the product. But consumers cannot know the exact value of the product, so before purchasing a product, consumers would evaluate possible regrets, that is, anticipated regret. Consumers’ anticipated regret would affect the demand for products, thereby affecting the profits of all parties. The manufacturer is the follower in the market, if the profit distribution is uneven, manufacturer raises the price to protect profits. Such fairness concerns inevitably lead to changes in prices and market demand.

Under the above background, this is a two-stage complete information game, in which both sides adjust their decisions according to the decisions of the other side to obtain maximum profits. Meanwhile, the retailer is as a dominant player to make decisions first. This fits the logic of how the Stackelberg game is construct. So in order to explore the impact of anticipated regret, fairness concerns and discounts on the supply chain, a Stackelberg game model is constructed. Then for coordinating the negative impact of equity concerns on the supply chain, the revenue sharing contract is designed to coordinate the supply chain. Based on the research conclusions, relevant suggestions are proposed for management practices.

In recent years, many scholars had introduced anticipated regret into the supply chain, and their studies focused on the impact on consumer behavior and the pricing decisions of enterprises. Bleichrodt et al. [1] introduced the abstract concept of regret into the actual model and first proposed the quantitative description of consumer regret. In the pre-sale situation, Nasiry et al. [2] found if the valuation was ultimately lower than the price paid, consumers’ early purchases triggered regret. Delayed purchases also led to regrets due to missed discounts or out of stock. Companies could provide refunds or allow resale to reduce the negative impact of regret. Youngwook [3] studied the consumers’ anticipated regret when facing the technological upgrades, and analyzed how the differences between expected utility and actual utility affected consumers’ decision-making in the next cycle. Daniel and Rebecca [4] explored the consumer’s aversion to uncertainty caused by regret from the perspective of psychology. Gao et al. [5] studied the impact of consumers’ anticipated regret on remanufactured products price under three power structures models. Liu et al. [6] analyzed the corporate price discrimination and profits under the duopoly game model with considering the consumer anticipated regret. Chen and Wang [7] introduced regret into the theory of multi-product dynamic pricing and found that consumers’ rational expectations depended on the retailer’s information transparency. Zhang et al. [8] analyzed the impact of anticipated regret on product innovation by constructing three power structure supply chain game models. The research showed that even if the anticipated regret existed, innovative products remained attractive. Besides, no leadership structure was more beneficial to consumers. Gao et al. [9] studied that the higher the sensitivity of consumer’s anticipated regret, the higher the level of innovation and the demand for remanufactured products. Zou et al. [10] studied that consumers’ anticipated regret increased the profits when products were optimized for design, the quality difference between the products was greater. Lim et al. [11] showed that prevention-centered consumers were more likely to become regret minimizers, and promotion-centered consumers were more likely to become most effective. Feng et al. [12] studied the impact of anticipated regret on the closed-loop supply chain, and the results showed that anticipated regret could ease the competition between new products and remanufactured products.

Many researches on fairness concerns of supply chain members mainly focused on decision-making and coordination. Katok and Pavlov [13] pointed out that the supply chain channel was inefficient because of the fairness concerns, bounded rationality and incomplete information. Tony and Paola [14] studied in a dual-channel supply chain how the corporate fairness concerns influenced pricing decisions. They analyzed the factors that affected the degree of fairness concerns of enterprises and how different distribution channels achieved fair distribution. Nie and Du [15] studied the pricing decisions of retailers with two kinds of fairness concerns, one was the retailers had concerns for the manufacturer’s revenue and another was the wholesale price obtained by another retailer, and they proved that under this circumstance the profits could not be coordinated through quantity discount contracts. Niu et al. [16] studied the importance of the power structure and fairness concerns in the supply chain and explored how they influenced the manufacturers’ market strategies. Qin et al. [17] introduced fairness concerns into supply chain coordination. Du et al. [18] coordinated the supply chain by designing wholesale price contracts, repurchase contracts and revenue-sharing contracts with considering fairness concerns in the newsboy model. Chen et al. [19] studied that the different fairness concerns degrees influenced the proportion of revenue sharing, the high-level fairness concerns increased the proportion of revenue sharing. Liu et al. [20] analyzed the impact of corporate price discrimination strategies on consumer surplus and social welfare with consumers’ fairness concerns. The results showed that when consumers’ fairness concerns increased, only retailers used price discrimination strategies would reduce the profits of manufacturers, when both manufacturers and retailers adopted price discrimination strategies, it would increase the profits and consumer’s surplus of manufacturers and retailers at the same time. Lin et al. [21] studied the manufacturers’ fairness concerns’ impact on the product price under the two different conditions (information symmetry or information asymmetry). Under the information symmetry, the optimal selling price remained unchanged, and when the information was asymmetry, fairness concerns increased the optimal price. Cao et al. [22] distinguished the coordination of revenue-sharing contracts with considering retailers’ and manufacturers’ fairness concerns, the results showed that manufacturers’ fairness concerns were detrimental to the closed supply chain. Wang et al. [23] studied the irrational fairness concern behavior of the e-commerce platform in E-CLSC, and the revenue-sharing was designed to realize system coordination with two cost-sharing contracts. Zhong et al. [24] showed that when two competing retailers had fairness concerns, the fairness concern behavior induced by peers always made the supplier always beneficial and detrimental to the retailer. Sarkar et al. [25] proposed a constant wholesale price contract could coordinate a decentralized channel in a manufacturer-led CLSC if the retailer’s advantageous inequality aversion was sufficiently strong. Kang et al. [26] were concerned about the impact of government subsidies and fairness concerns on the decision-making and coordination involved in the poverty alleviation supply chain (PASC) composed of a farmer enterprise and a core enterprise.

According to the above-mentioned literatures, the existing literature only considers the anticipated regret or fairness concerns unilaterally. No research has taken into consideration both the consumer’s anticipated regret and the manufacturer’s fairness concerns in a retailer-led supply chain.

The content is distributed as follows. The problem is described in Section 2. Centralized and decentralized decision models are discussed in Section 3. A profit-sharing contract is designed in Section 4. A numerical simulation is to prove the correctness of the above conclusions in Section 5. The conclusion is summarized in Section 6.

## 2. Problem description

In order to compare and analyze the impact of various factors on the price, demand and profit of all parties more conveniently. The symbol definitions and assumptions in the model are shown in Table 1.

**Table 1:** Parameters and definitions.

Parameters	Definition	Range
*c*	Manufacturer’s product costs	*c* = 0
*w*	Wholesale price per unit	
*p*_*o*_	The original price per unit	
*p*_*d*_	The discount price per unit	
*d*	Discount rate	0.5<*d*<1
*θ*_*H*_	Consumers with a higher preference for discount product	
*θ*_*L*_	Consumers with a lower preference for discount product	
*f*	Fairness concern coefficient	0<*f*<1
*r*	Consumer heterogeneity	
*γ*	Regret sensitivity coefficient	0<*γ*<1

A retailer-led supply chain consists of a manufacturer and a retailer. In order to attract customers, retailers adopt a strategy of discount sales. The product cost is *c*, and the wholesale price is *w*. Retailer independently determines the original price *p*_*o*_ and discount price *p*_*d*_, *p*_*d*_ = *p*_*o*_*d* (*d* is the discount rate), 0.5<*d*<1. Consumers are classified based on acceptance of discount price products, *θ*_*H*_ represents consumers with high preference, *θ*_*L*_ represents consumers with low preference, 0<*θ*_*L*_<*θ*_*H*_≤1. Let *θ*_*H*_ = 1, *θ*_*L*_ = 1−*r*, *r* represents consumer heterogeneity. *r* = *θ*_*H*_−*θ*_*L*_. Assume that the probabilities that consumers are high preference and low preference are 1/2 respectively. The utility of the original price product is *v*, and *v*∈[0,1] obeys uniform distribution.

Whether the consumers choose to accept the discount price product, there is a 1/2 probability to regret. When the consumers purchase the discount price product, the expected utility is expressed as $v\frac{\theta_{H}+\theta_{L}}{2}$. Because consumers have anticipated regret, it would reduce the utility, according to the research by Jiang [27], anticipated regret’s negative utility can be expressed as:

$$A.R=-\gamma k(U_{f}-U_{c})$$ (1)

Where *γ* represents the regret sensitivity coefficient, 0<*γ*<1, *U*_*f*_ represents the utility that consumers give up purchasing the products, *U*_*c*_ represents the utility that consumers purchase the products. *k* represents the possibility that the utility of giving up buying a certain product is greater than the utility of buying another product, in this case, consumers would regret it. According to the consumer preference types in the previous article, the probability of consumer’s regret is *k* = 1/2. In the situation of purchasing original price products, consumers with high preference *θ*_*H*_ would regret it, and the utility of purchasing the discount price product is *U*_*f*_ = *θ*_*H*_*v*−*p*_*o*_*d*, while the utility of purchasing the original price products is *U*_*c*_ = *v*−*p*_*o*_. Similarly, in the situation of purchasing discount price products, consumers with low preference *θ*_*L*_ would regret it. The utility of purchasing the original price product is *U*_*f*_ = *v*−*p*_*o*_, while the utility of purchasing the discount price products is *U*_*c*_ = *θ*_*L*_*v*−*p*_*o*_*d*. In summary, with considering the anticipated regret, the consumers’ net utility of purchasing different price products can be expressed:

$$U_{o}=v-\frac{1}{2}p_{o}\left(2+(1-d)\gamma \right)$$ (2)

$$U_{d}=\frac{1}{2}(v(2-r-r\gamma )+p_{o}(\gamma -d(2+\gamma )))$$ (3)

When *U*_*o*_>0 and *U*_*o*_>*U*_*d*_, consumers choose to give up discounts. When *U*_*d*_>0 and *U*_*d*_>*U*_*o*_, consumers choose to purchase discount price product. The demand functions can be obtained respectively:

$$D_{o}=\int_{\begin{array}{c} U_{o}>U_{d}\\ U_{o}>0 \end{array}}f(v)dv=\{\begin{array}{ll} 1-\frac{(2+\gamma )p_{o}-\gamma dp_{o}}{2} & if\;p_{o}\leq \frac{2}{2+\gamma -d\gamma }\\ 1+\frac{2p_{o}(-1+d)}{r} & if\;\frac{2}{2+\gamma -d\gamma }\leq p_{o}\leq \frac{r}{2-2d}\\ 0 & if\;\frac{r}{2-2d}\leq p_{o} \end{array}$$ (4)

$$D_{d}=\int_{\begin{array}{c} U_{d}>U_{o}\\ U_{d}>0 \end{array}}f(v)dv=\{\begin{array}{ll} 0 & if\;p_{o}\leq \frac{-2+r+r\gamma }{\gamma -d(2+\gamma )}\\ \frac{p_{o}(4(-1+d)+r(2+\gamma -d\gamma ))}{r(-2+r+r\gamma )} & if\;\frac{-2+r+r\gamma }{\gamma -d(2+\gamma )}\leq p_{o}\leq \frac{r}{2-2d}\\ 1-\frac{p_{o}((2+\gamma )d-\gamma )}{1+(1-r+\gamma -r\gamma )-\gamma } & if\;p_{o}\geq \frac{r}{2-2d} \end{array}$$ (5)

Assume that $A=\frac{d-1}{r}$ and $B=\frac{d(2+\gamma )-\gamma }{2-r-r\gamma }$ for ease of calculation, then the demand functions can be simplified as:

$$D_{o}=1+2p_{o}A$$ (6)

$$D_{d}=-p_{o}(2A+B)$$ (7)

It is supposed that the products are homogeneous whether it is discounted or not. The quantity of products is unlimited.

Then the profit functions of two parties can be get respectively:

$$\pi_{NR}=(p_{o}-w)(1+2p_{o}A)-(p_{o}d-w)p_{o}(2A+B)$$ (8)

$$\pi_{NM}=(w-c)(1-Bp_{o})$$ (9)

## 3. Models of different decision-making

### 3.1 Centralized decision-making model with manufacturer’s fairness neutrality

In centralized decision-making model (represented by subscript C), which have a common goal to maximize the overall profits, the overall profits function of model C is:

$$\pi_{}^{C}=\pi_{NR}+\pi_{NM}=p_{o}(1+2Ap_{o}-(2A+B)w)+(dp_{o}-w)p_{o}(-2A-B)$$ (10)

According to the expressions of *A* and *B*, it can be gotten easily that *A*<0, *B*>0, then find the point of maximum profits that satisfies $\frac{\partial^{2}\pi_{}^{C}}{\partial p_{o}{}^{2}}=4A-2(2A+B)d<0$. From function(10), the optimal retail price $p_{o}^{C}{}_{}^{*}$ is:

$$p_{o}^{C}{}_{}^{*}=\frac{1+Bc}{4A(-1+d)+2Bd}$$ (11)

Substituting $p_{o}^{C}{}_{}^{*}$ into the function(10), the optimal overall profits *π*^C^* can be obtained:

$$\pi_{}^{C}{}_{}^{*}=\frac{{\left(1+Bc\right)}^{2}}{8A(-1+d)+4Bd}-c$$ (12)

Simultaneously the $D_{o}^{C}{}_{}^{*}$ and $D_{d}^{C}{}_{}^{*}$ are respectively obtained as follows:

$$D_{o}^{C}{}_{}^{*}=1+\frac{2A(1+Bc)}{4A(-1+d)+2Bd}$$ (13)

$$D_{d}^{C}{}_{}^{*}=\frac{\left(1+Bc)(-2A-B\right)}{4A(-1+d)+2Bd}$$ (14)

Proposition 1: $p_{o}^{C}{}_{}^{*}$ is negatively related to *γ*, and $D_{o}^{C}{}_{}^{*}$ is positively related to *γ*, $D_{d}^{C}{}_{}^{*}$ is negatively related to *γ*, and *π*^C^* is positively related to *γ*.

Proof: Let functions (11), (12), (13), and (14) take the derivative of *γ* respectively, and the results are: $\frac{\partial p_{o}^{C}{}_{}^{*}}{\partial \gamma }<0$, $\frac{\partial D_{o}^{C}{}_{}^{*}}{\partial \gamma }>0$, $\frac{\partial D_{d}^{C}{}_{}^{*}}{\partial \gamma }<0$, $\frac{\partial \pi_{}^{C}{}_{}^{*}}{\partial \gamma }>0$.

Proposition 1 shows that in model C the anticipated regret has a great impact on consumers’ decision-making. Because the revenue of increasing sales is greater than the loss of falling price, in other words, the overall profits increase. And consumers are more inclined to buy original price product with less risk. When consumers are more sensitive to regret, they make decisions more carefully. In order to promote consumer, retailers often choose to lower product prices. This proposition suggests that companies in the supply chain should do a full investigation when selecting target markets. Moreover, discount rates and product prices are determined by taking into account the sensitivity of consumer’s anticipated regret.

Proposition 2: $p_{o}^{C}{}_{}^{*}$ is positively related to *r*; $D_{o}^{C}{}_{}^{*}$ is positively related to *r*; $D_{d}^{C}{}_{}^{*}$ is negatively related to *r*.

Proof: Let functions (11), (12), (13), and (14) take the derivative of *d* respectively, and the results are: $\frac{\partial p_{o}^{C}{}_{}^{*}}{\partial r}>0$, $\frac{\partial D_{o}^{C}{}_{}^{*}}{\partial r}>0$, $\frac{\partial D_{d}^{C}{}_{}^{*}}{\partial r}<0$.

Proposition 2 shows that when consumer heterogeneity increases, product price would rise, while consumers tend to give up discounts. Because the first derivative form of function (12) is too complicated to judge the correlation directly, the consumer heterogeneity’s impact on the overall profits is analyzed in the following numerical analysis.

### 3.2 Retailer-led decentralized decision-making model with the manufacturer’s fairness concerns

In the retailer-led decentralized decision-making model (represented by subscript RL), the retailer first determines the retail price, then the manufacturer decides the wholesale price according to the retail price. Since the product cost does not affect the conclusion of this article, it is assumed that *c* = 0. According to the above descriptions and the profit functions (8) and (9), the new profit functions in Model RL can be expressed as follows:

$$\pi_{R}^{RL}=\pi_{NR}=-w+p_{o}(1+Bw)+p_{o}{}^{2}(-2A(-1+d)-Bd)$$ (15)

$$\pi_{M}^{RL}=\pi_{NM}-f\pi_{NR}=fp_{o}(Bdp_{o}-1-2A(1-d)p_{o})+(1+f)(1-Bp_{o})w$$ (16)

First, the reaction function of *w* with respect to *p*_*o*_ can be obtained by performing a first-order derivative of function (16), it is $w=\frac{1}{B}+c-p_{o}$. Then $w=\frac{1}{B}+c-p_{o}$ is substituted into the function (15) to get the new expression about profits:

$$\pi_{R}^{RL}{}_{}^{*}=w+p_{o}(1+Bw)+p_{o}{}^{2}(-2A(-1+d)-Bd)$$ (17)

Then the optimal retail price $p_{o}^{RL}{}_{}^{*}$ is:

$$p_{o}^{RL}{}_{}^{*}=\frac{B(-3+2f(-1+d))+4Af(-1+d)}{-2B(B+2A(-1+f)+Bf)+2B(2A+B)(-1+f)d}$$ (18)

Substituting $p_{o}^{RL}{}_{}^{*}$ into $w=\frac{1}{B}+c-p_{o}$, the optimal wholesale price *w*^RL^* is obtained as:

$$w_{}^{RL}{}_{}^{*}=\frac{\left(4A(-1+d)+B(-1+2d))(-B-3Bf+2(2A+B)(-1+d)f^{2}\right)}{2B^{2}(B(-1+d(-1+f)-f)(1+f)+2A(-1+d)(-1+f^{2}))}$$ (19)

Substituting $p_{o}^{RL}{}_{}^{*}$ and *w*^RL^* into the functions (15) and (16), the optimal profits of both parties can be obtained:

$$\pi_{R}^{RL}{}_{}^{*}=\frac{B(8A(-1+d)+B(-5+4d))+8B(2A+B)(-1+d)f-4{\left(2A+B\right)}^{2}{\left(-1+d\right)}^{2}f^{2}}{4B^{2}(B(-1+d(-1+f)-f)(1+f)+2A(-1+d)(-1+f^{2}))}$$ (20)

$$\begin{array}{l} \pi_{M}^{RL}{}_{}^{*}=\frac{\left(\begin{array}{l} B{\left(4A+B-2(2A+B)d\right)}^{2}+3B(2A+B)(-1+d)(B+8A(-1+d)+4Bd)f-\\ 8{\left(2A+B\right)}^{2}{\left(-1+d\right)}^{2}(B+2A(-1+d)+Bd)f^{2}+4{\left(2A+B\right)}^{3}{\left(-1+d\right)}^{3}f^{3} \end{array}\right)}{4B^{2}{\left(-B(1+d)+2A(-1+d)(-1+f)+B(-1+d)f\right)}^{2}} \end{array}$$ (21)

Substituting $\pi_{R}^{RL}{}_{}^{*}$ and $\pi_{M}^{RL}{}_{}^{*}$ into the demand functions (6) and (7), the optimal demand functions can be obtained:

$$D_{o}^{RL}{}_{}^{*}=1+\frac{-3AB+2A(2A+B)(-1+d)f}{-B(B+2A(-1+d)+Bd)+B(2A+B)(-1+d)f}$$ (22)

$$D_{d}^{RL}{}_{}^{*}=\frac{\left(2A+B)(3B-2(2A+B)(-1+d)f\right)}{-2B(B+2A(-1+d)+Bd)+2B(2A+B)(-1+d)f}$$ (23)

The overall profits *π*^RL^* is:

$$\pi_{}^{RL}{}_{}^{*}=\pi_{R}^{RL}{}_{}^{*}+\pi_{M}^{RL}{}_{}^{*}$$ (24)

Based on the above analysis, the following three propositions can be obtained.

Proposition 3: $p_{o}^{RL}{}_{}^{*}$, $w_{}^{RL}{}_{}^{*}$, $D_{d}^{RL}{}_{}^{*}$, $\pi_{R}^{RL}{}_{}^{*}$ and $\pi_{M}^{RL}{}_{}^{*}$ are all negatively related to *γ*, while $D_{o}^{RL}{}_{}^{*}$ is positively related to *γ*.

Proof: Let functions (18)–(23) take the derivative of *γ* respectively, and the results are: $\frac{\partial p_{o}^{RL}{}_{}^{*}}{\partial \gamma }<0$, $\frac{\partial w_{}^{RL}{}_{}^{*}}{\partial \gamma }<0$, $\frac{\partial D_{d}^{RL}{}_{}^{*}}{\partial \gamma }<0$, $\frac{\partial \pi_{R}^{RL}{}_{}^{*}}{\partial \gamma }<0$, $\frac{\partial \pi_{M}^{RL}{}_{}^{*}}{\partial \gamma }<0$, $\frac{\partial D_{o}^{RL}{}_{}^{*}}{\partial \gamma }>0$.

Proposition 3 shows that as consumers become more sensitive to regret, the retail price and wholesale price both gradually decline, and the profits of both parties also gradually decline, but the demand for original price product gradually increases, while the demand for discount price product gradually decreases. The two main reasons for this trend are as follows. First, when the price of the original price product increases, consumers who are highly sensitive to price would be afraid that prices would rise in the future, and they would regret if they do not buy now, so the demand increases. Second, because consumers are warier of “regret”, they would be more cautious in the face of discount price products. Assuming that the product is a necessity, it has a lower elasticity of demand, then with the increase of regret, the demand is roughly same, manufacturer’s costs are the same, but the retail price and wholesale price have fallen, so that the manufacturer’s profit falls. In addition, because the manufacturer is the follower according to the retailer’s decision, the retail price has fallen even more. That means the profits of both parties would decrease. This proposition suggests that retailers should carefully choose the price discount strategy.

Proposition 4: $D_{o}^{RL}{}_{}^{*}$ is positively related to *r*, and $D_{d}^{RL}{}_{}^{*}$ is negatively related to *r*.

Proof: Let functions (22) and (23) take the derivative of *r* respectively, and the results are: $\frac{\partial D_{o}^{RL}{}_{}^{*}}{\partial r}>0$, $\frac{\partial D_{d}^{RL}{}_{}^{*}}{\partial r}<0$.

Proposition 4 shows that as consumer heterogeneity increases, consumers’ demand for original price product increases, while the demand for discount price product decreases. This is because when the heterogeneity of consumers gradually increases, there would be great differences in the degree of preference for discounted products among consumer groups. That means consumers’ recognition of the value of discount price product varies. When consumers understand that there is heterogeneity between consumer groups, they would not lightly adopt the opinions of other consumers when purchasing discount price products. However, they more carefully estimate the value of the product and the possibility of regret after purchasing it. Therefore, consumers would be more inclined to buy original price product.

Proposition 5: $p_{o}^{RL}{}_{}^{*}$, *w*^RL^* are positively related to *f*. $D_{d}^{RL}{}_{}^{*}$, $D_{o}^{RL}{}_{}^{*}$, $\pi_{R}^{RL}{}_{}^{*}$, $\pi_{M}^{RL}{}_{}^{*}$, and *π*^RL^* are all negatively related to *f*.

Proof: Let functions (18)–(24) take the derivative of *f* respectively, and the results are: $\frac{\partial p_{o}^{RL}{}_{}^{*}}{\partial f}>0$, $\frac{\partial w_{}^{RL}{}_{}^{*}}{\partial f}>0$, $\frac{\partial D_{d}^{RL}{}_{}^{*}}{\partial f}<0$, $\frac{D_{o}^{RL}{}_{}^{*}}{\partial f}<0$, $\frac{\partial \pi_{R}^{RL}{}_{}^{*}}{\partial f}<0$, $\frac{\partial \pi_{M}^{RL}{}_{}^{*}}{\partial f}<0$, $\frac{\partial \pi_{}^{RL}{}_{}^{*}}{\partial f}<0$.

According to proposition 5, as the fairness concerns increase, the prices both increase, and consumers’ demand for both original and discount price product gradually declines. Retailer’s profits, manufacturer’s profits, and the overall profits would decline. When the manufacturer’s fairness concerns increase, the manufacturer would seek fairness at the expense of its profits. For example, the manufacturer would choose to increase the wholesale price in order to increase its profits and seek fair distribution of profits in the supply chain. Then, in order to maintain the profits, the retailer chooses to increase the retail price because of the increased cost, and the consumers’ demand would naturally decrease. For the retailer, if the profits increase by increasing prices is less than the profits lost due to lower demand, the profits decrease. For the manufacturer, if the revenue gained by increasing the wholesale price is less than the revenue lost due to lower demand, the profits decrease. Therefore, the overall profits have fallen. This reminds companies in the supply chain that they should not ignore the existence of fairness concerns. To reduce damage that the fairness concerns may cause to the supply chain, manufacturer and retailer can adopt cooperation, such as formulating a revenue-sharing contract.

## 4. Coordination with a revenue-sharing contract

In the retailer-led supply chain game model (Model RS), the revenue-sharing contract is used to coordinate profit distribution (represented by subscript as RS). The content of the revenue-sharing contract is as follows. The retailer asks the manufacturer for the lower wholesale price while providing part of the sales revenue with the manufacturer. When the retailer obtains a lower wholesale price, the retailer could lower the sales price to increase sales, thereby improving the overall profits. Both parties jointly negotiate to determine the revenue-sharing ratio *φ*, 0<*φ*<1.

The profit functions after the revenue-sharing contract can be obtained as:

$$\pi_{R}^{RS}=((1-\varphi )p_{o}-w)(1+2p_{o}A)+((1-\varphi )p_{o}d-w)p_{o}(-2A-B)$$ (25)

$$\begin{array}{c} \pi_{M}^{RS}=(w-c)(1-Bp_{o})+\varphi (p_{o}(1+2p_{o}A)-dp_{o}^{2}(2A+B))-\\ f(\left(p_{o}-w)(1+2p_{o}A)-(p_{o}d-w)p_{o}(2A+B\right)) \end{array}$$ (26)

The retailer’s profit represents the remaining profits after sharing part of the sales revenue. The manufacturer’s profit is composed of three parts, the first part is the profits after wholesaling to the retailer. The second part is the proportional revenue obtained from the revenue-sharing contract. The third part is the negative effects of fairness concerns.

There are two goals in a revenue-sharing contract: The first one is making the overall profits after contract coordination closer to that in Model C and to make the optimal retail prices in the two models equal. The second one is to make the profits of retailer and manufacturer after coordination not lower than before.

First, the response function of *w* about *p*_*o*_ after coordination can be obtained as:

$$w_{}^{RS}=\frac{1+4A(-1+d)p_{o}(f-\varphi )+\varphi +Bp_{o}(-1+(-1+2d)f-2d\varphi )}{B(1+f)}$$ (27)

Substituting *w*^*RS*^ to $\pi_{R}^{RS}$, $p_{o}^{RS*}$ after coordination can be obtained.


$$p_{o}^{RS*}=\frac{4A(-1+d)(f-\varphi )+B(-3-2d\varphi +f(-2+2d+\varphi ))}{2B(2A(-1+d)(-1+f)(1+\varphi )+B(-1-f+d(-1+f)(1+\varphi )))}$$ (28)

When $p_{o}^{RS*}$ is equal to the $p_{o}^{C*}$, the coordination role for the supply chain can be realized.


$$\frac{4A(-1+d)(f-\varphi )+B(-3-2d\varphi +f(-2+2d+\varphi ))}{2B(2A(-1+d)(-1+f)(1+\varphi )+B(-1-f+d(-1+f)(1+\varphi )))}=\frac{1+Bc}{4A(-1+d)+2Bd}$$ (29)

Therefore:

$$\begin{array}{l} f^{RS*}=\left(\begin{array}{c} B(B(-1+2d)(1+d\varphi )+2A(-1+d)(-1-\varphi +2d\varphi ))+\\ \left(8A(-1+d)\varphi +B(6+4d\varphi ))A(-1+d\right) \end{array}\right)/\\ \;\left(\begin{array}{l} B(-1+d)((2A+B)(-1+2d)-2A\varphi )+\\ 2(2(2A+B)(-1+d)+B\varphi )A(-1+d) \end{array}\right) \end{array}$$ (30)

It can be seen that when *f* = *f*^RS^*, the optimal retail price is the same in Model C or in Model RS. Taking $p_{o}^{RS*}$ into the function(27), *w*^RS^* can be gotten as:

$$w^{RS*}=\frac{\left(\begin{array}{l} B^{2}\left(\begin{array}{l} 4d^{2}{\left(f-\varphi \right)}^{2}+(1+f)(1+f(2+\varphi )-2\varphi )\\ -2d(f^{2}(3-\varphi )+{\left(1-\varphi \right)}^{2}+f(3-5\varphi )) \end{array}\right)+16A^{2}{\left(-1+d\right)}^{2}{\left(f-\varphi \right)}^{2}\\ +4AB(-1+d)(-1+2\varphi +(-1+4d)\varphi^{2}+f^{2}(-3+4d+\varphi )+f(-3+(5-8d)\varphi )) \end{array}\right)}{2B^{2}(1+f)(2A(1-d)(1-f)(1+\varphi )-B(1+f+d(1-f)(1+\varphi )))}$$ (31)

Then the optimal profits of both parties can be obtained.


$$\pi_{R}^{RS*}=\frac{\left(\begin{array}{l} 16{\left(A(1-d)(f-\varphi )\right)}^{2}+8AB(1-d)\left(\begin{array}{l} 1+2f(1+(1-d)f)+\\ \varphi (1+f(4-4d+f)-(1-2d)\varphi ) \end{array}\right)+\\ B^{2}\left(\begin{array}{l} 5+f(8+f{\left(2-\varphi \right)}^{2}-10\varphi )+4{\left(d(f-\varphi )\right)}^{2}-4\varphi +\\ 4d(\varphi +f(4+f)\varphi -\varphi^{2}-1-2f(1+f)) \end{array}\right) \end{array}\right)}{4B^{2}(1+f)(B(1+f+d(1-f)(1+\varphi ))-2A(1-d)(1-f)(1+\varphi ))}$$ (32)

$$\begin{array}{l} \pi_{M}^{RS*}=\left(\begin{array}{l} -2B^{2}c(2+f)(-2A(-1+d)(-2+f+f\varphi )+B(f-d(-2+f+f\varphi )))\\ (-4A(-1+d)(-2+\varphi +f\varphi )+B(-3+\varphi +f\varphi -2d(-2+\varphi +f\varphi )))+\\ \left(2+f)(f-\varphi )(4A(-1+d)(f-\varphi )+B(-3+\varphi -2d\varphi +f(-2+2d+\varphi ))\right)\\ \left(\begin{array}{l} 8A^{2}{\left(-1+d\right)}^{2}(f-\varphi )+2AB(-1+d)(1+4(-1+d)f+\varphi -(4d+f)\varphi )+\\ B^{2}(2f+d(1+2(-2+d)f+\varphi -(2d+f)\varphi )) \end{array}\right)+\\ (1+f)\left(\begin{array}{l} -4A(-1+d)(-2+\varphi +f\varphi )+\\ B(-3+\varphi +f\varphi -2d(-2+\varphi +f\varphi )) \end{array}\right)\\ \left(\begin{array}{l} -16A^{2}{\left(-1+d\right)}^{2}{\left(f-\varphi \right)}^{2}-4AB(-1+d)\left(\begin{array}{l} -2+f(-2+(-3+4d)f)+\varphi +\\ f(6-8d+f)\varphi +(-1+4d)\varphi^{2} \end{array}\right)+\\ B^{2}\left(\begin{array}{l} -4d^{2}{\left(f-\varphi \right)}^{2}+f(-1-2f+(3+f)\varphi )+\\ 2d(2+2f+3f^{2}-(1+f(6+f))\varphi +\varphi^{2}) \end{array}\right) \end{array}\right) \end{array}\right)/\\ \left(4B^{2}(2+f){\left(-Bf+2A(-1+d)(-2+f+f\varphi )+Bd(-2+f+f\varphi )\right)}^{2}\right) \end{array}$$ (33)

To make the profits after coordination are not less than before, the following two conditions need to be met.


$$\pi_{R}^{RL}\leq \pi_{R}^{RS}$$ (34)

$$\pi_{M}^{RL}\leq \pi_{M}^{RS}$$ (35)

Solve the above two inequalities jointly. The range of *φ* is obtained.


$$\begin{array}{l} \frac{\left(2A(-1+d)+Bd)(\begin{array}{l} B(8A(-1+d)+B(-5+4d))+\\ 8B(2A+B)(-1+d)f-4{\left(2A+B\right)}^{2}{\left(-1+d\right)}^{2}f^{2} \end{array}\right)}{B^{2}(B(-1+d(-1+f)-f)(1+f)+2A(-1+d)(-1+f^{2}))}\\ <\varphi <1-\left((2A(-1+d)+Bd)(\begin{array}{l} B{\left(4A+B-2(2A+B)d\right)}^{2}+\\ 3B(2A+B)(-1+d)(B+8A(-1+d)+4Bd)f-\\ 8{\left(2A+B\right)}^{2}{\left(-1+d\right)}^{2}(B+2A(-1+d)+Bd)f^{2}+\\ 4{\left(2A+B\right)}^{3}{\left(-1+d\right)}^{3}f^{3} \end{array})\right)/\\ B^{2}{\left(2A(-1+d+f-df)+B(1+d+f-df)\right)}^{2} \end{array}$$ (36)

Proposition 5: The fairness concern coefficient satisfies function (30), and the revenue-sharing ratio satisfies function (36), supply chain coordination can be achieved.

## 5. Numerical analysis

MATLAB is used to analyze the impact of anticipated regret, the fairness concerns coefficient, the price discount coefficient and the consumer heterogeneity on the supply chain.

### 5.1 Sensitivity analysis of regret sensitive coefficient

Suppose *d* = 0.88, *r* = 0.6, *c* = 1, *f* = 0.2, *γ*∈[0.1].

**Fig 1:**
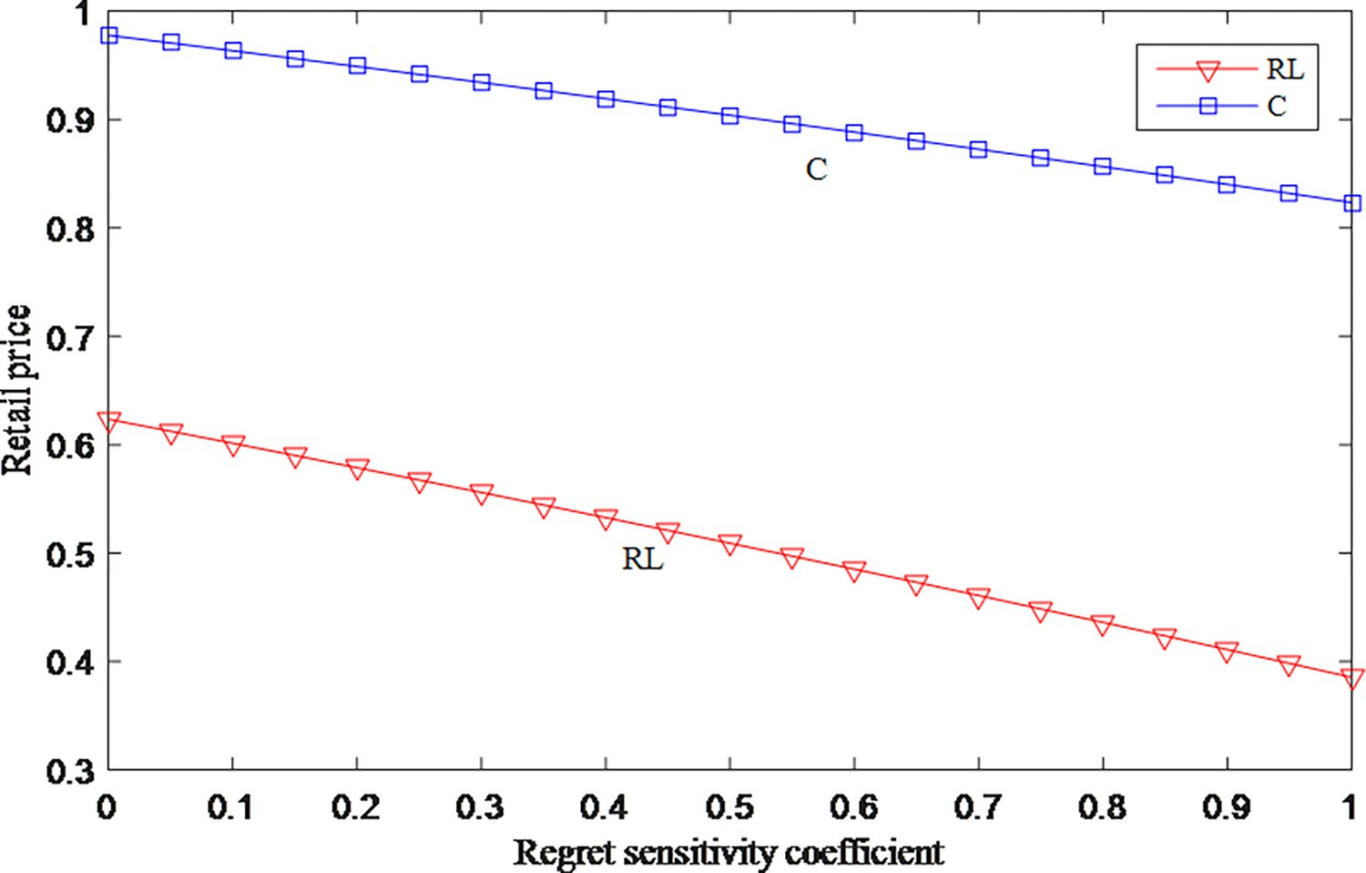
The impact of anticipated regret on retail price.

**Fig 2:**
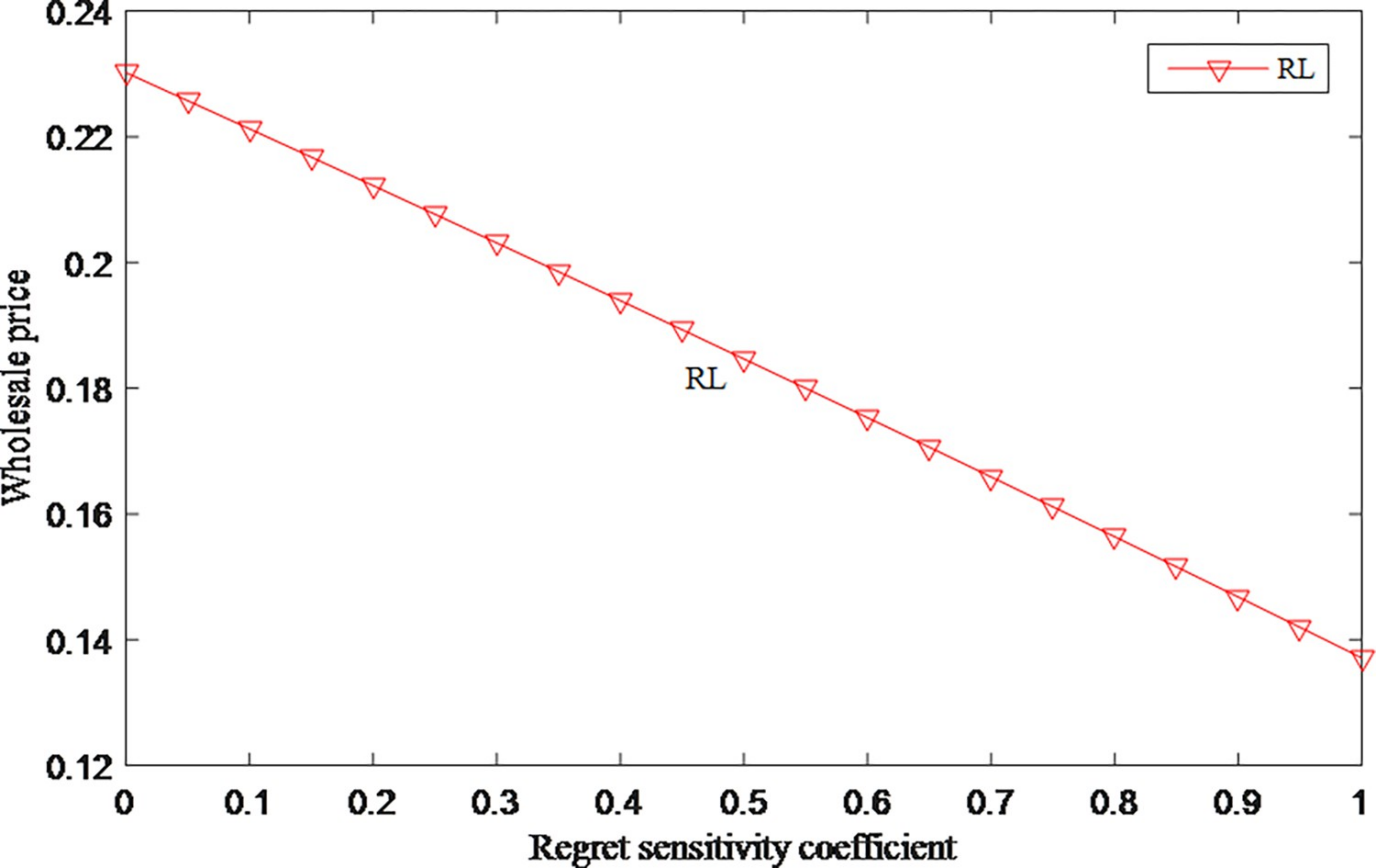
The impact of anticipated regret on wholesale price.

The relationships among regret sensitivity coefficient, retail price and wholesale price are shown in Figs 1 and 2, the retail price and wholesale price both decrease as consumers feel more sensitive to regret. When consumers in the market have high levels of anticipated regret, the retailer and manufacturer tend to lower the price. This shows that consumers are relatively cautious when they have anticipated regret. Lower prices are adopted to attract consumers.

**Fig 3:**
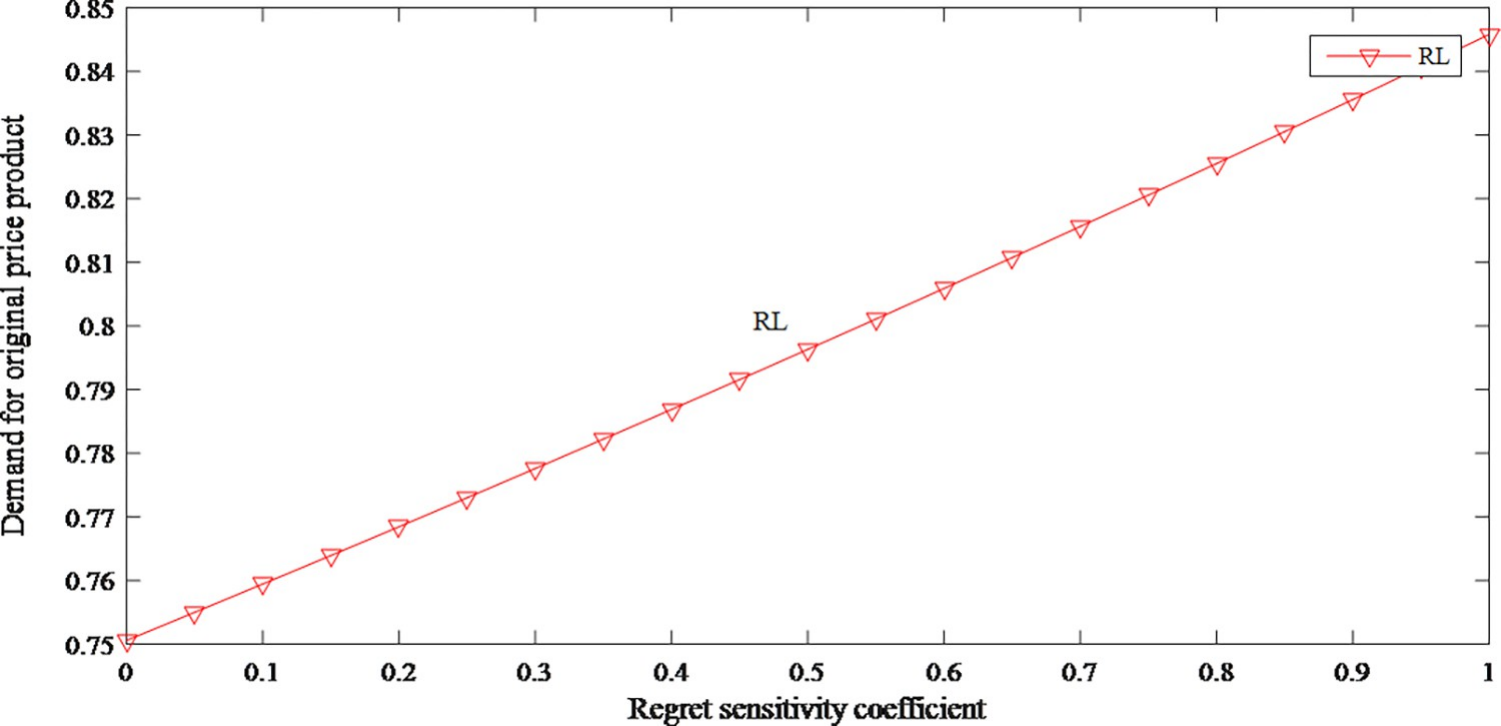
The impact of anticipated regret on demand for original price product.

**Fig 4:**
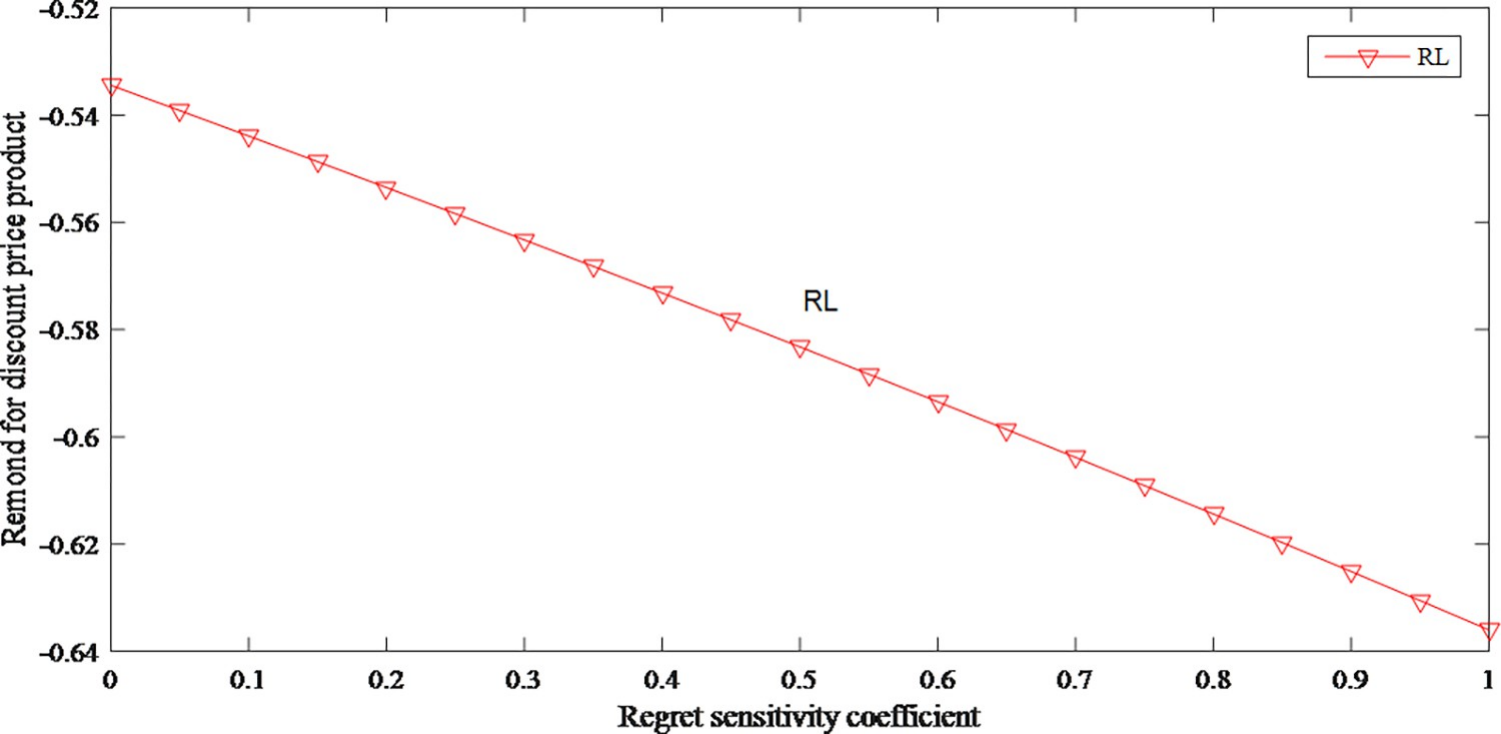
The impact of anticipated regret on demand for discount price product.

According to Figs 3 and 4, regret sensitivity has an opposite effect on the demand for both products. When consumers have high-level regret sensitivity, they would be more inclined to purchase original price product. When the price of original price product is close to consumers’ expectations, the attractiveness of discount price product further declines.

**Fig 5:**
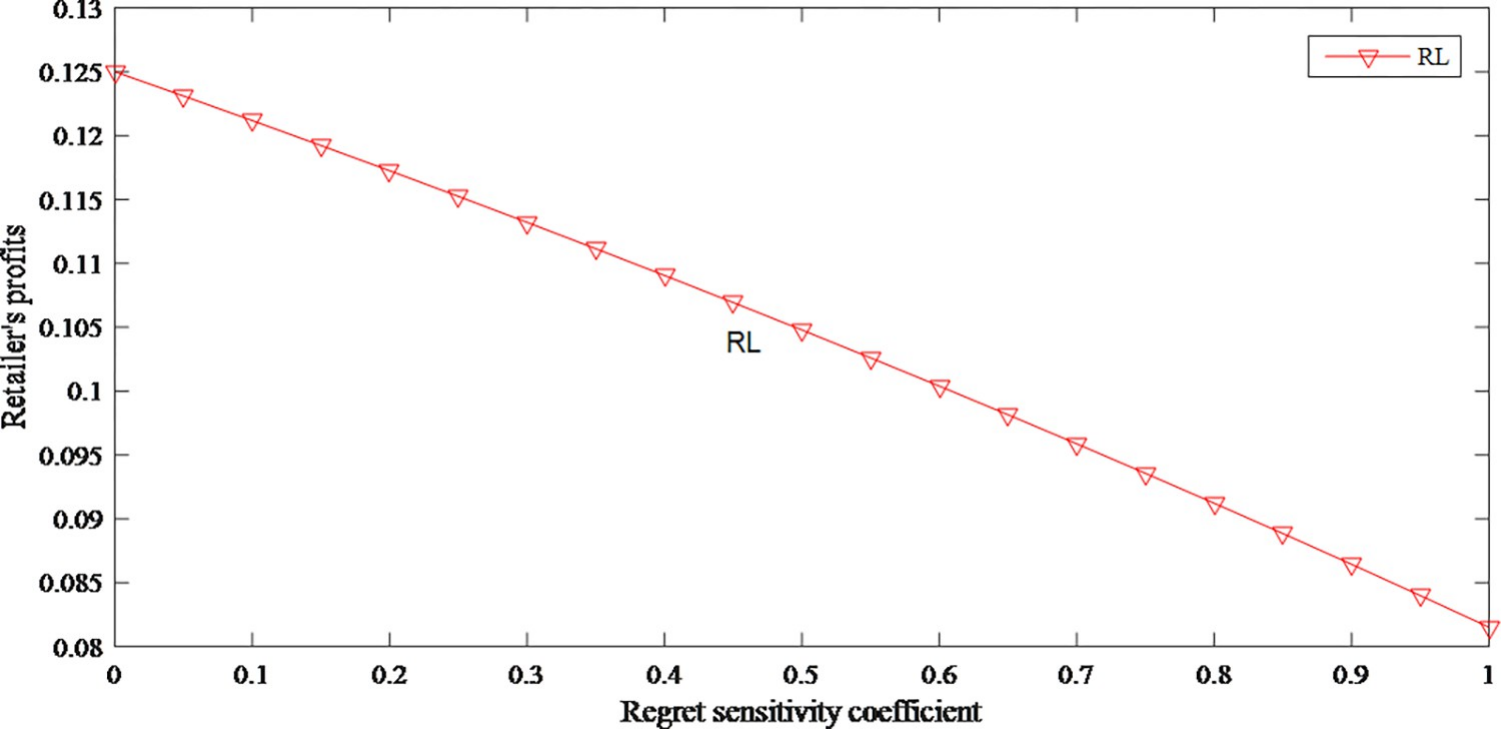
The impact of anticipated regret on retailer’s profits.

In model RL, due to “non-rational people” factors such as consumers’ anticipated regret and fairness concerns, the retailer’s profits show a downward trend as consumers’ sensitivity increases. The specific impact is shown in Fig 5.

**Fig 6:**
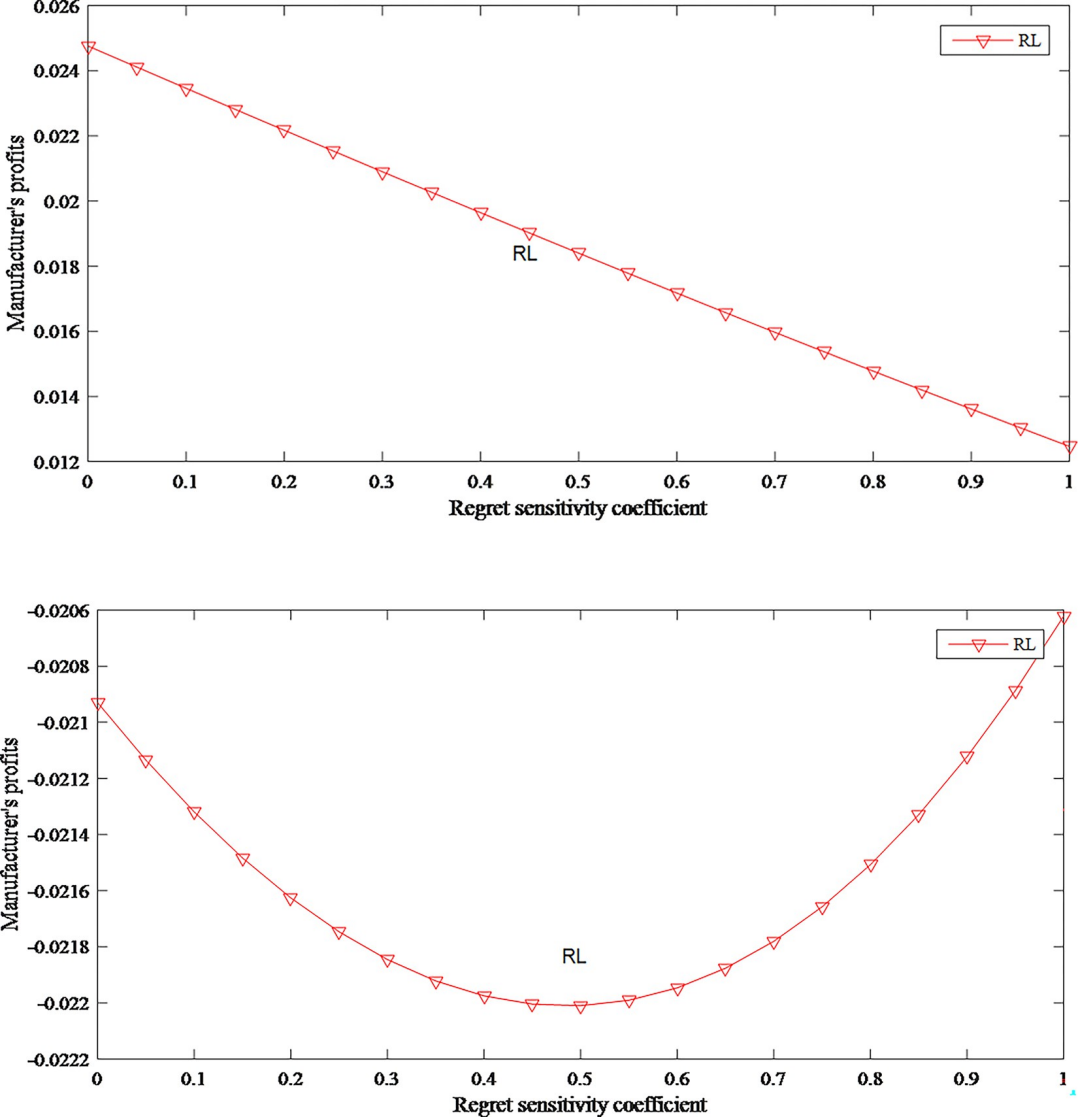
The impact of anticipated regret on manufacturer’s profits. (a) *f* = 0.2, (b) *f* = 0.9.

There are two different situations about the impact of regret sensitivity on the manufacturer’s profits. When the manufacturer has low-level fairness concerns, the profits decrease as the consumer regret sensitivity coefficient increases, as shown in Fig 6(A). On the contrary, when in the situation of high-level fairness concerns, the profit first increases and then decreases as the regret sensitivity coefficient increases, as shown in Fig 6(B). However, we can see that when the manufacturer has high-level fairness concerns, the manufacturer is in a state of loss. In order to reverse this trend, the manufacturer can pay more attention to the customer group with a high-level anticipated regret.

**Fig 7:**
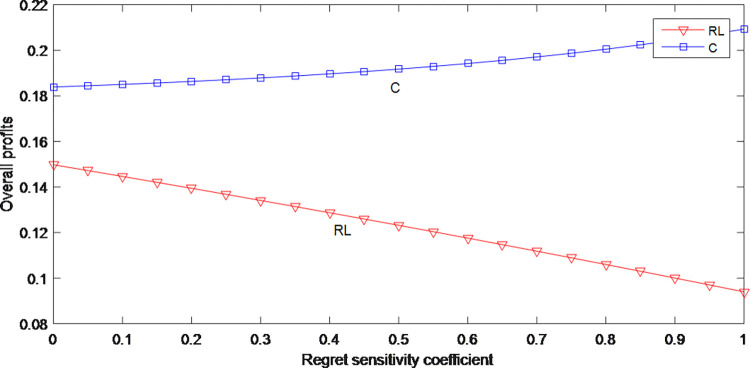
The impact of anticipated regret on the overall profits.

Fig 7 shows that the overall profits in model RL have a downward trend as the consumer regret sensitivity coefficient increases, which is the opposite of the situation in model C. It means if supply chain companies choose not to cooperate, their profits would be reduced due to the consumer’s anticipated regret. Therefore, when the consumer’s behavior is considered, the manufacturer and retailer should choose cooperation strategies to prevent their profits.

From Figs 1 and 7, it can be seen that whether in model C or RL, the higher the consumer’s regret sensitivity, the lower the price. But the price in model C is higher than in the model RL. Consumers’ regret has the opposite effect on the overall profits, and the overall profits in model C are always greater than the overall profits in model RL. When facing consumers with a high degree of regret, manufacturer and retailer should adopt a cooperative strategy to maintain higher retail price and overall profit, and to ensure that overall profit maintains an upward trend.

## 5.2 Sensitivity analysis of manufacturer’s fairness concerns

This paper intends to explore how the fairness concern coefficient influenced product prices, market demand and profits. Assume *d* = 0.88, *r* = 0.7, *c* = 1, *γ* = 0.2, *f*∈[0,1].

**Fig 8:**
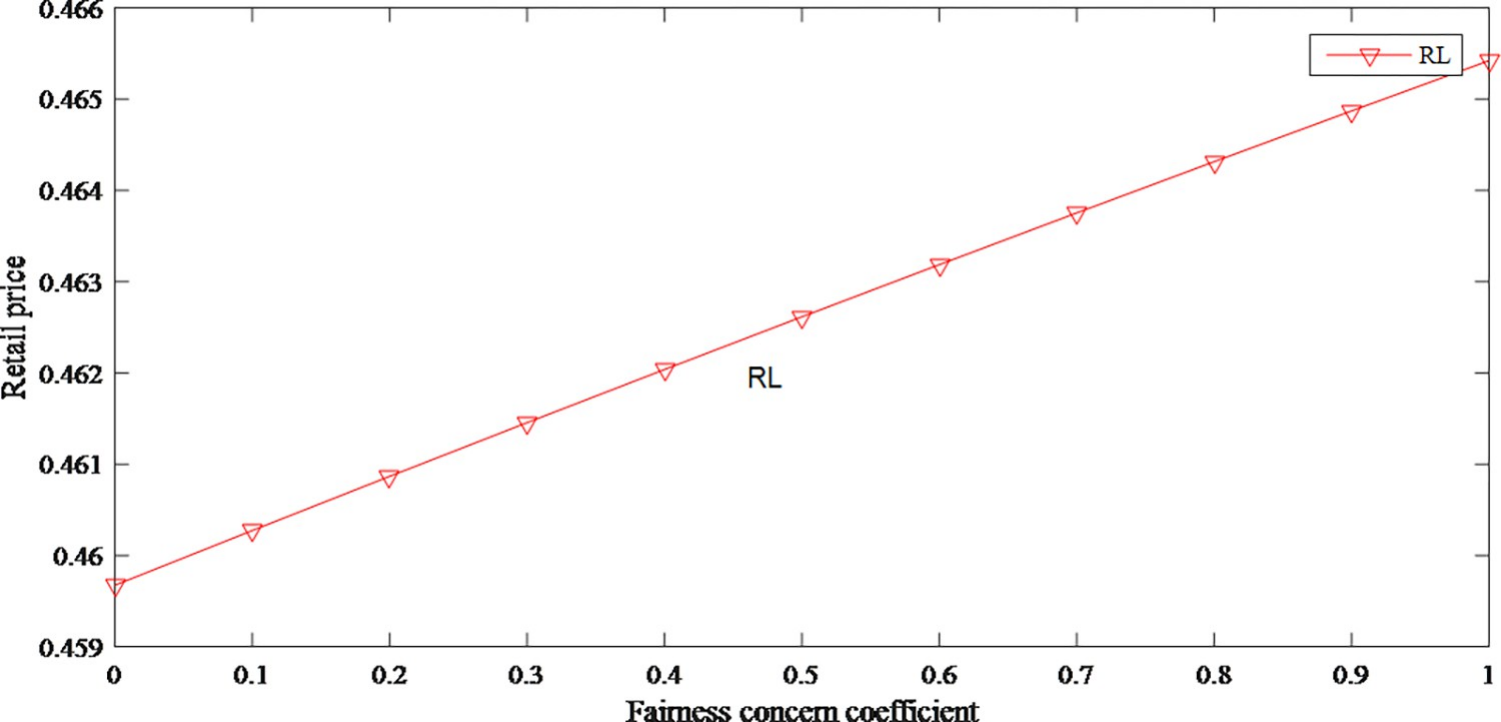
The impact of fairness concern on retail price.

**Fig 9:**
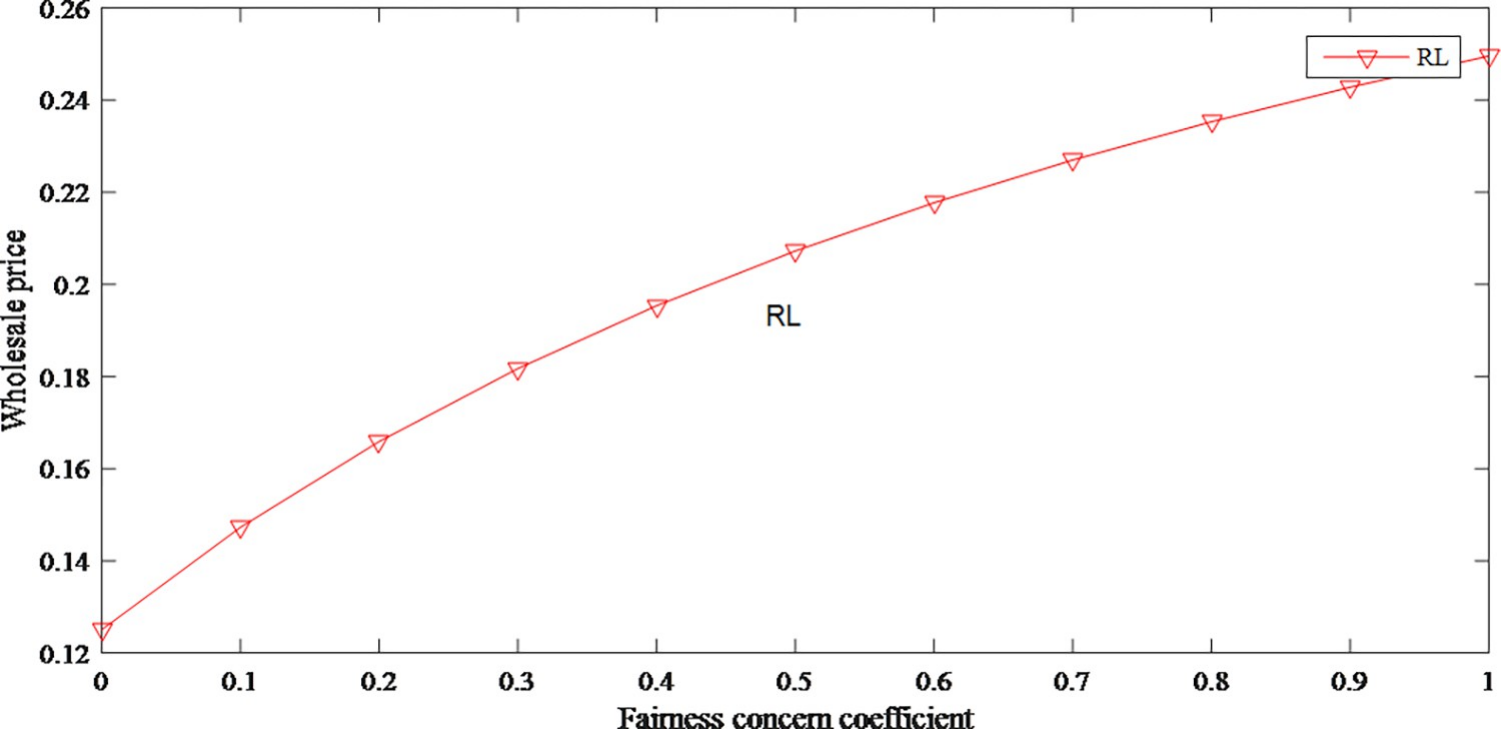
The impact of fairness concern on wholesale price.

In the retailer-led supply chain, the manufacturer has less power in profit distribution. They guarantee their profits by increasing the wholesale price. As the wholesale price has risen, the original price has also risen. Therefore, the fairness concerns have an upward effect on product price as shown in Figs 8 and 9.

**Fig 10:**
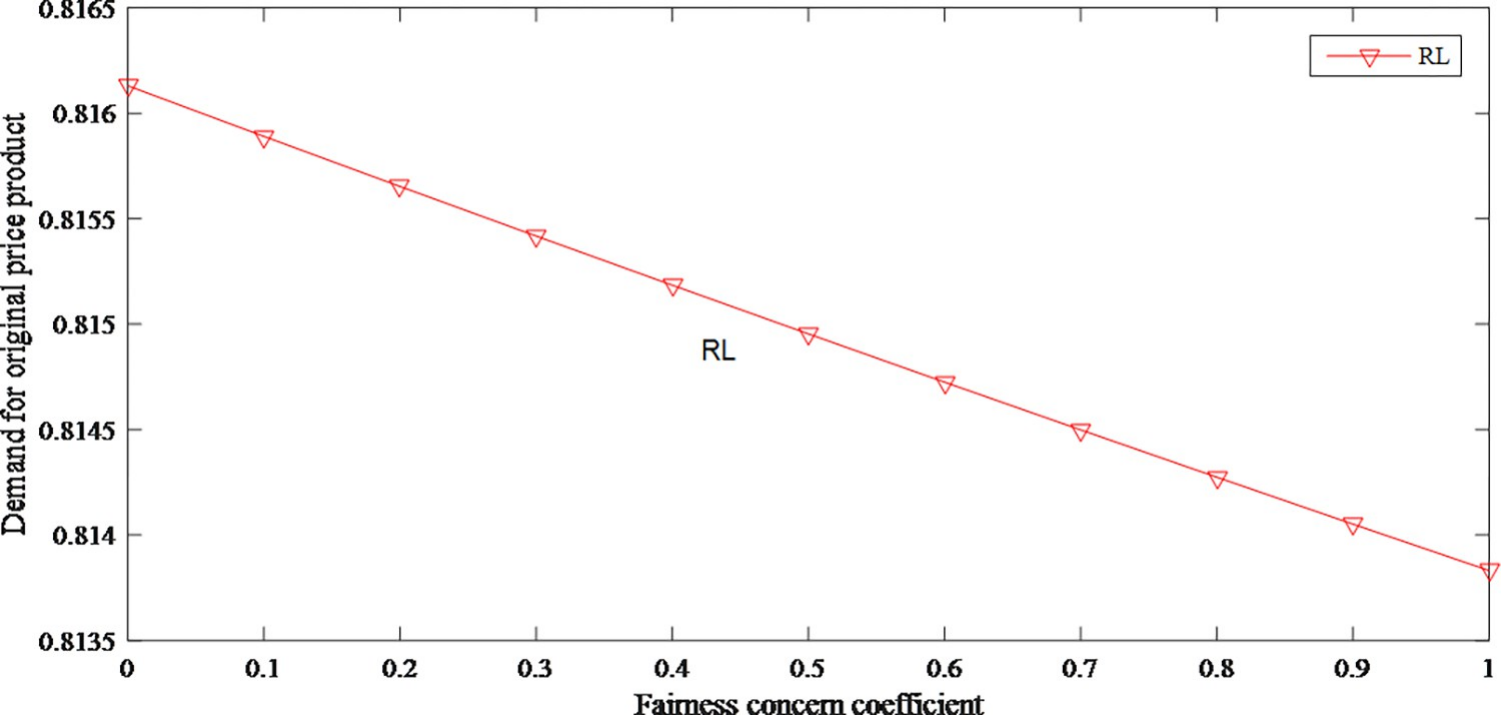
The impact of fairness concerns on demand for original price product.

**Fig 11:**
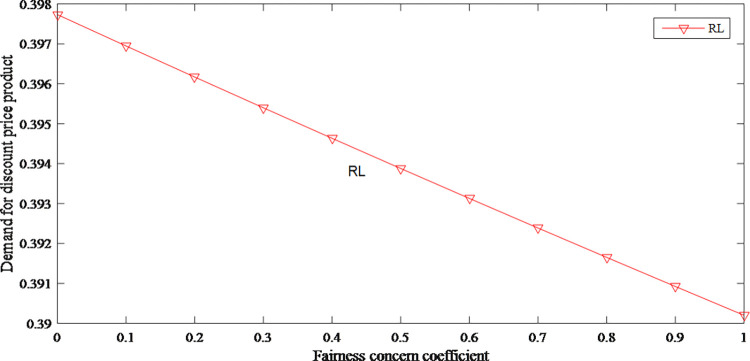
The impact of fairness concerns on demand for discount price product.

When the product prices increase, the market demand for original and discount price product has declined to different degrees. Therefore, the fairness concerns have shown a downward effect on demands as shown in Figs 10 and 11.

**Fig 12:**
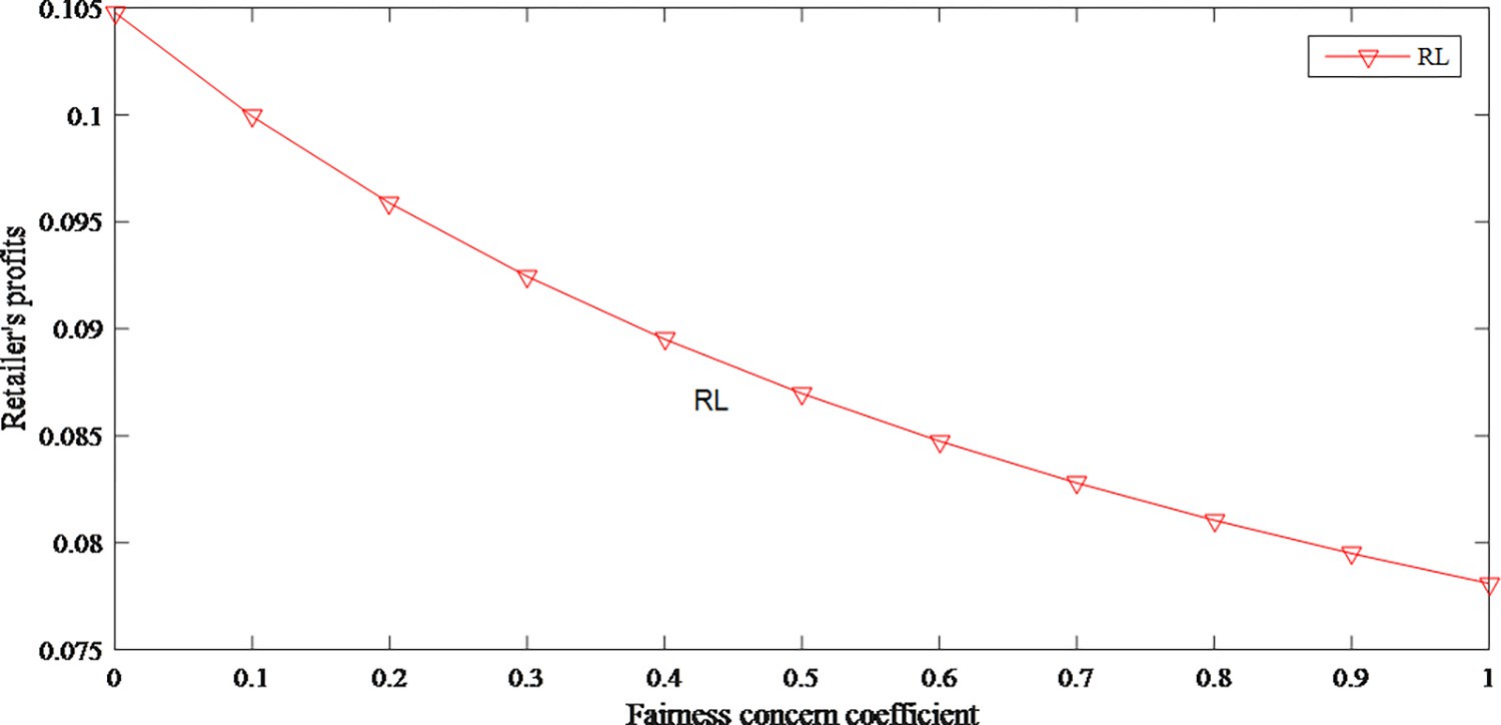
The impact of fairness concerns on retailer’s profits.

**Fig 13:**
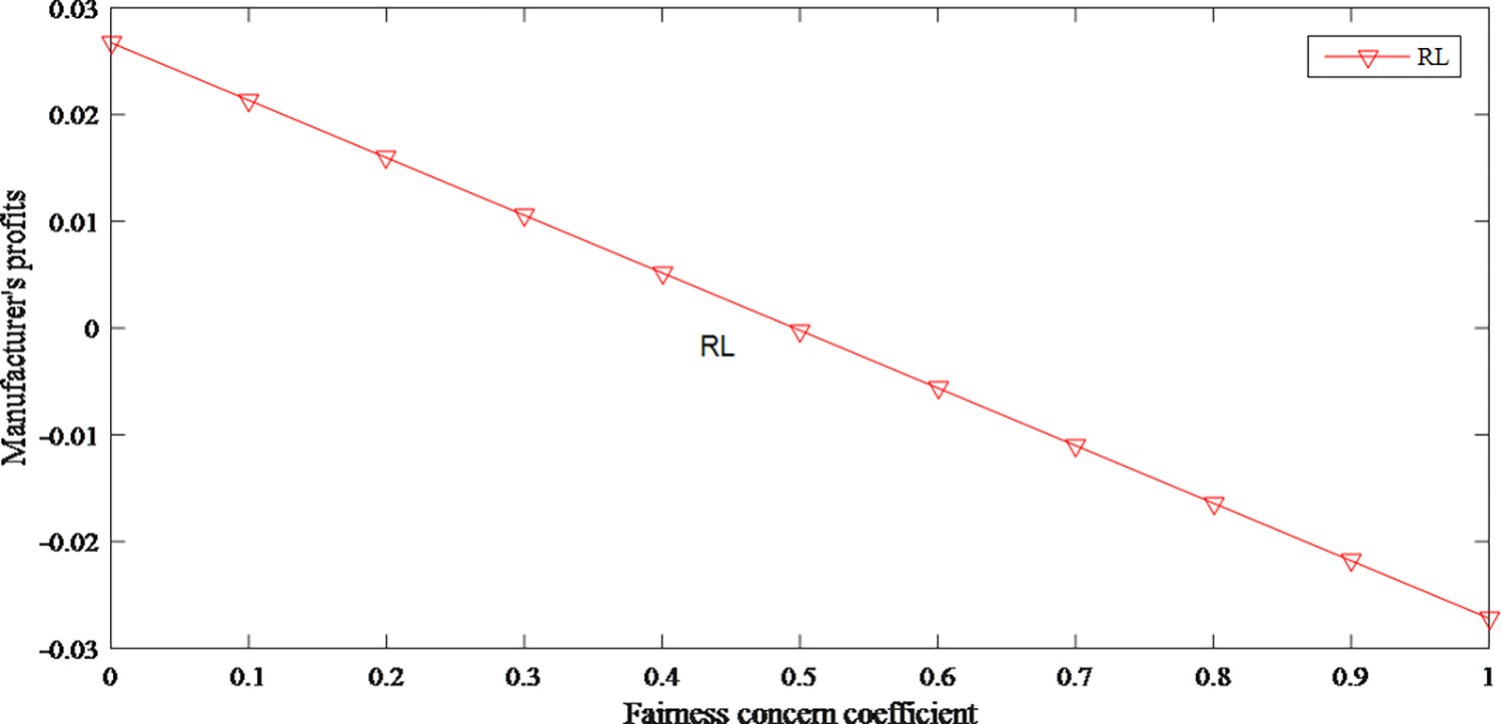
The impact of fairness concerns on manufacturer’s profits.

Figs 12 and 13 reflect the profits of the retailer and manufacturer decrease as the manufacturer’s fairness concerns increases. Moreover, compared with the retailer, manufacturer’s profits have fallen even more. The overall profits lead to decline because of too much attention to profit distribution.

### 5.3 Sensitivity analysis of consumer heterogeneity

**Fig 14:**
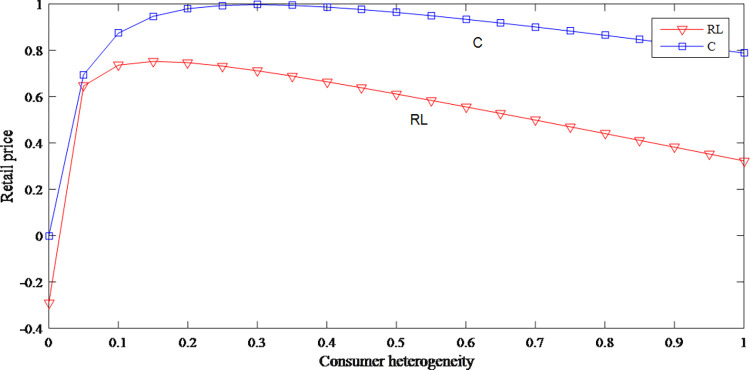
The impact of consumer heterogeneity on retail price.

**Fig 15:**
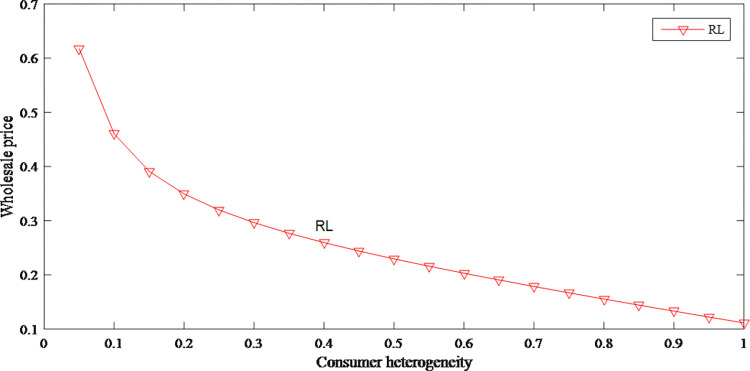
The impact of consumer heterogeneity on wholesale price.

Based on Figs 14 and 15, the impact of consumer heterogeneity on retail price is uncertain but has a negative effect on wholesale price. When the consumer heterogeneity is low, which means the gap in consumer’s acceptance of discount price product is small, the original price increases with the consumer heterogeneity increases. When the consumer heterogeneity continues to increase, the original price then decreases.

**Fig 16:**
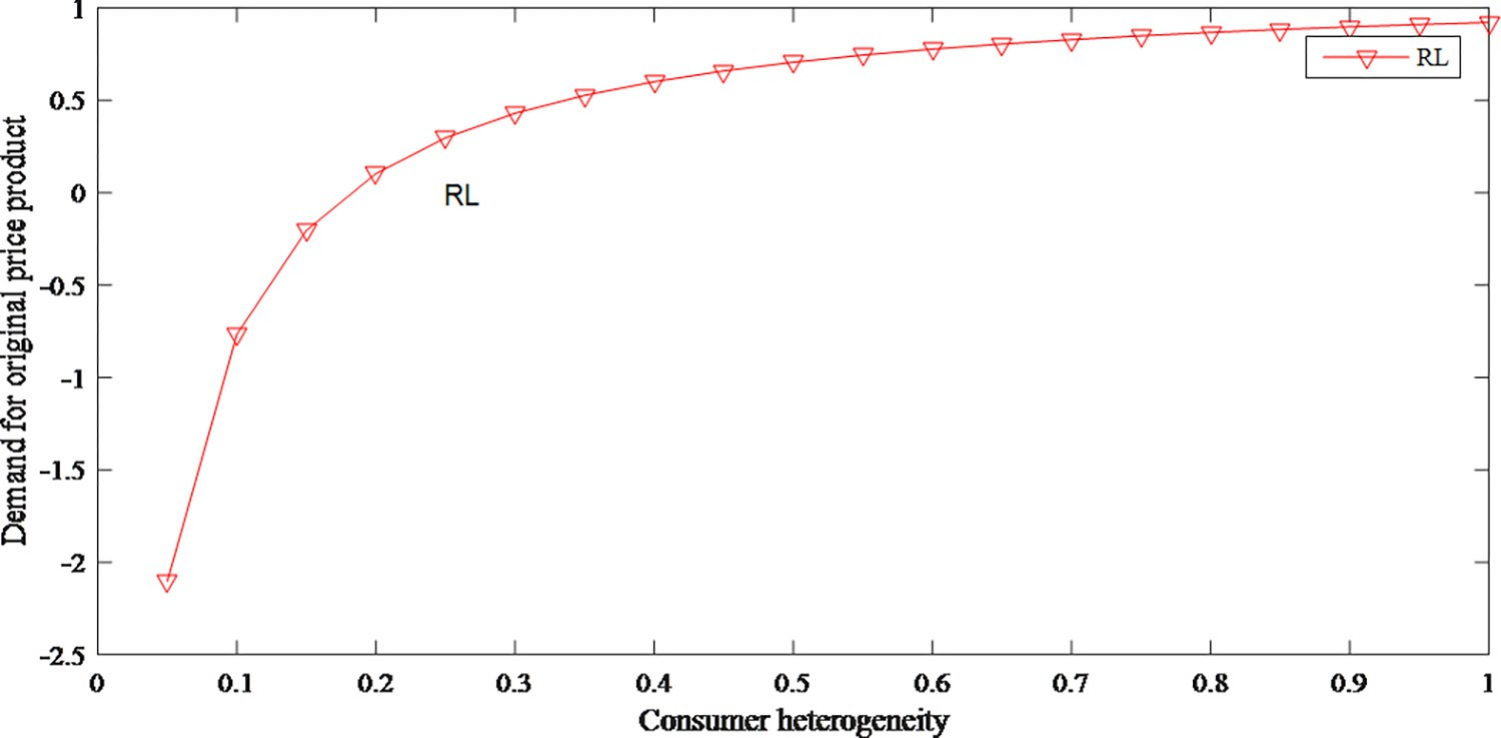
The impact of consumer heterogeneity on demand for original price product.

**Fig 17:**
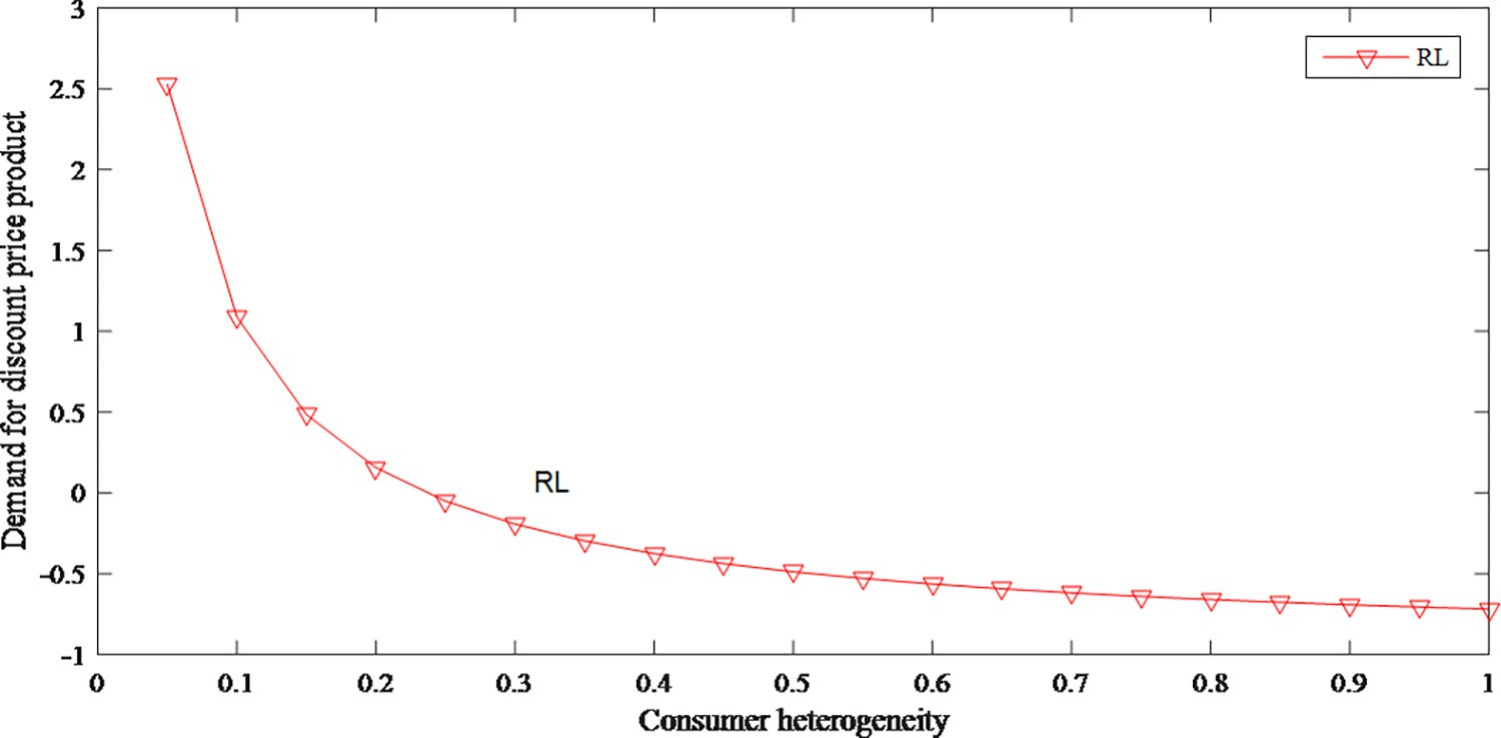
The impact of consumer heterogeneity on demand for discount price product.

**Fig 18:**
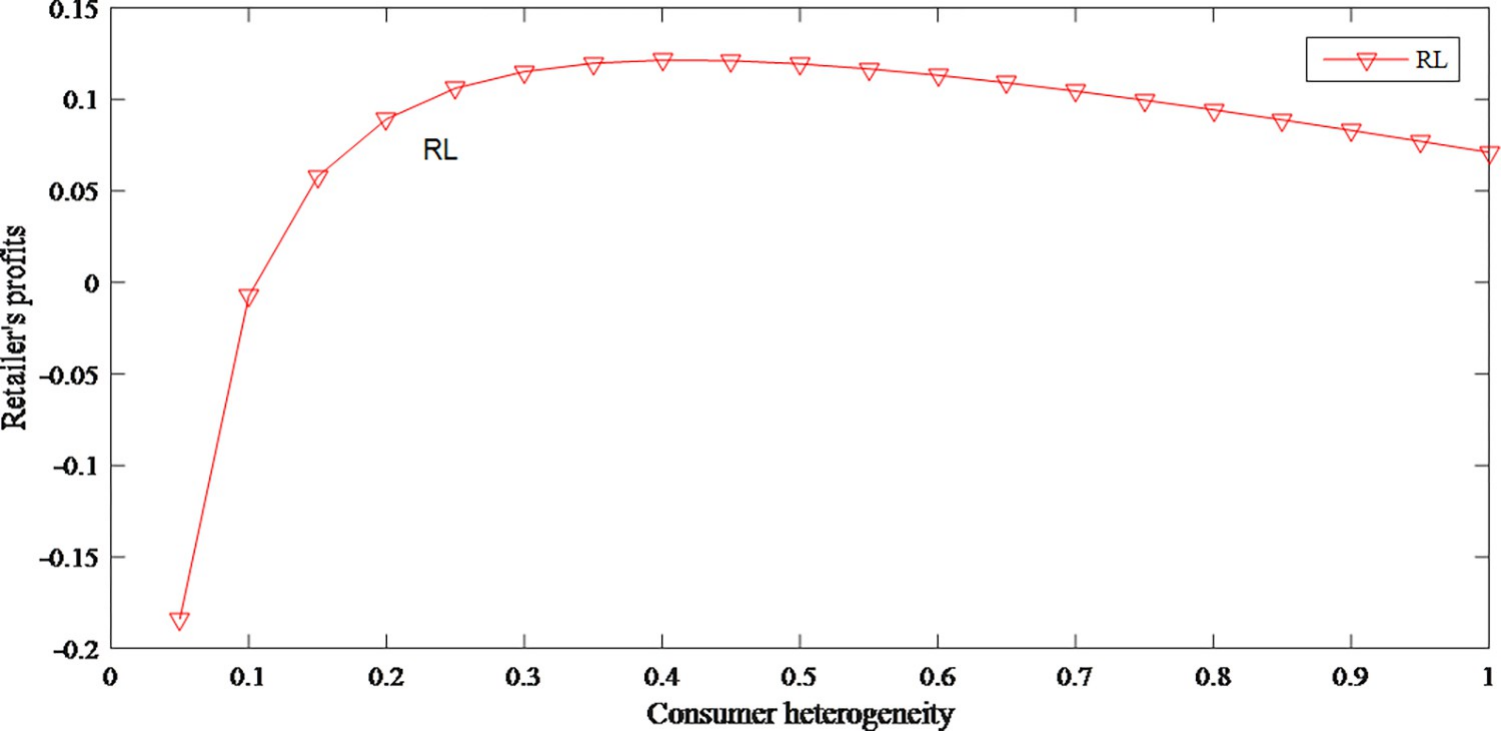
The impact of consumer heterogeneity on retailer’s profits.

It can be seen from Figs 16 and 17 that consumer heterogeneity has an opposite effect on the demand for different prices, which shows that consumer heterogeneity increases the uncertainty of the supply chain. Fig 18 reflects that when consumer heterogeneity is low, retailer’s profits increase as consumer heterogeneity increases. According to this, retailer can create different marketing strategies for different groups to improve the cognitive differences of consumer groups, thereby profits are effectively increased.

### 5.4 Sensitivity analysis of price discount rate

In real life, the discount rate of most commodities is more than 50%. The parameters are set as *r* = 0.6, *γ* = 0.3, *f* = 0.2, *d*∈(0.5,1).

**Fig 19:**
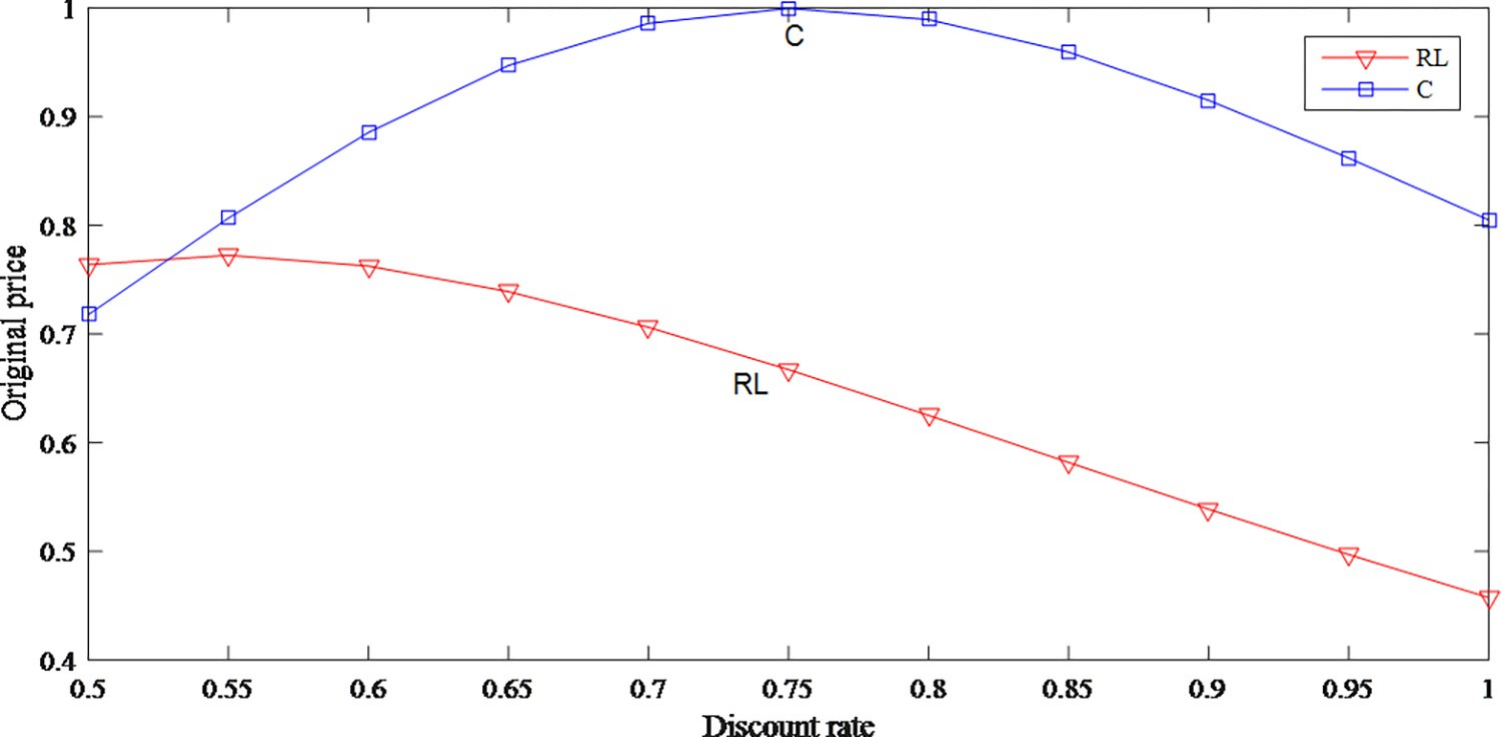
The impact of discount rate on original price.

**Fig 20:**
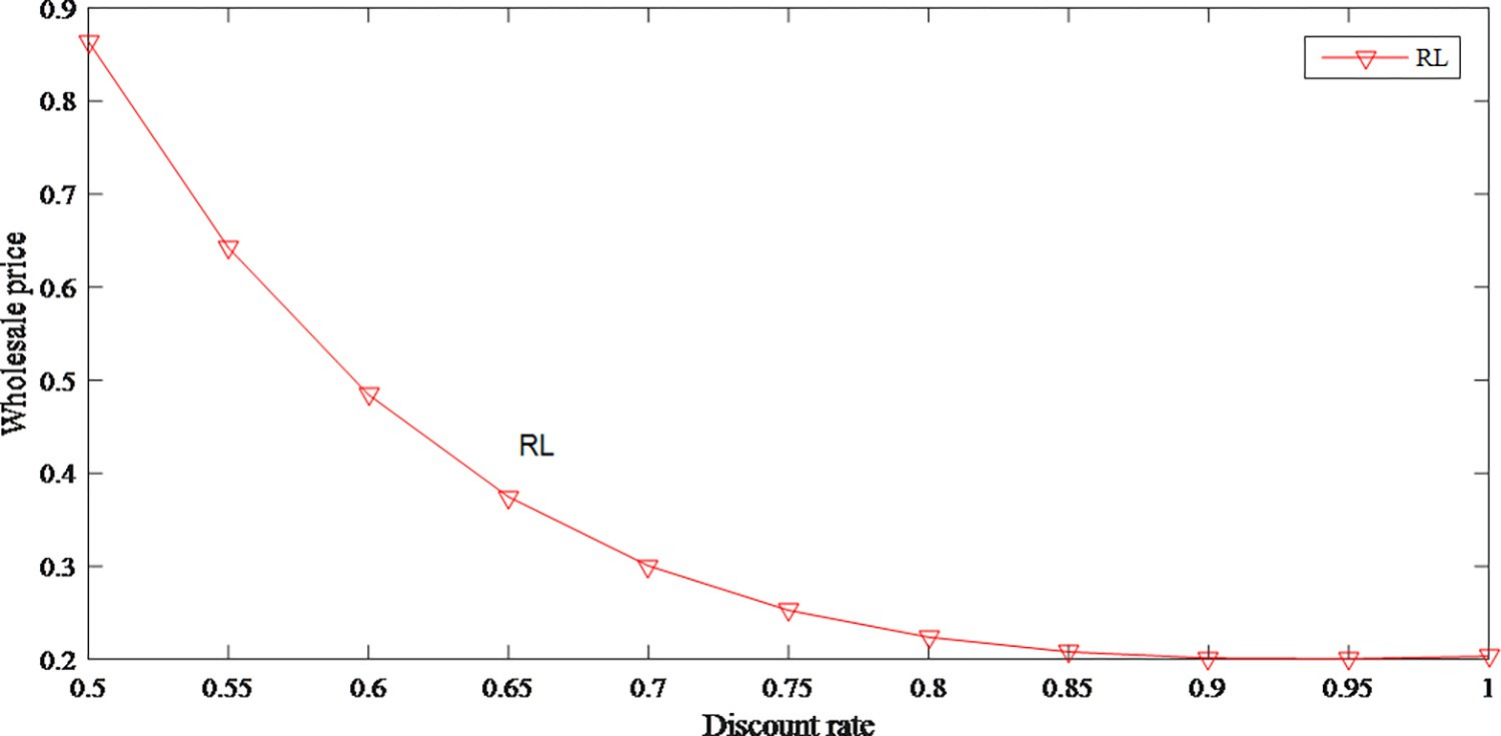
The impact of discount rate on wholesale price.

Figs 19 and 20 show the prices both decrease with the discount rate increases in the retailer-led model. When discount rate is high, in order to expand sales, retailer cannot price their products too high. According to the optimal response function of the manufacturer to the retailer, the wholesale price decreases according to the decrease of the retail price.

**Fig 21:**
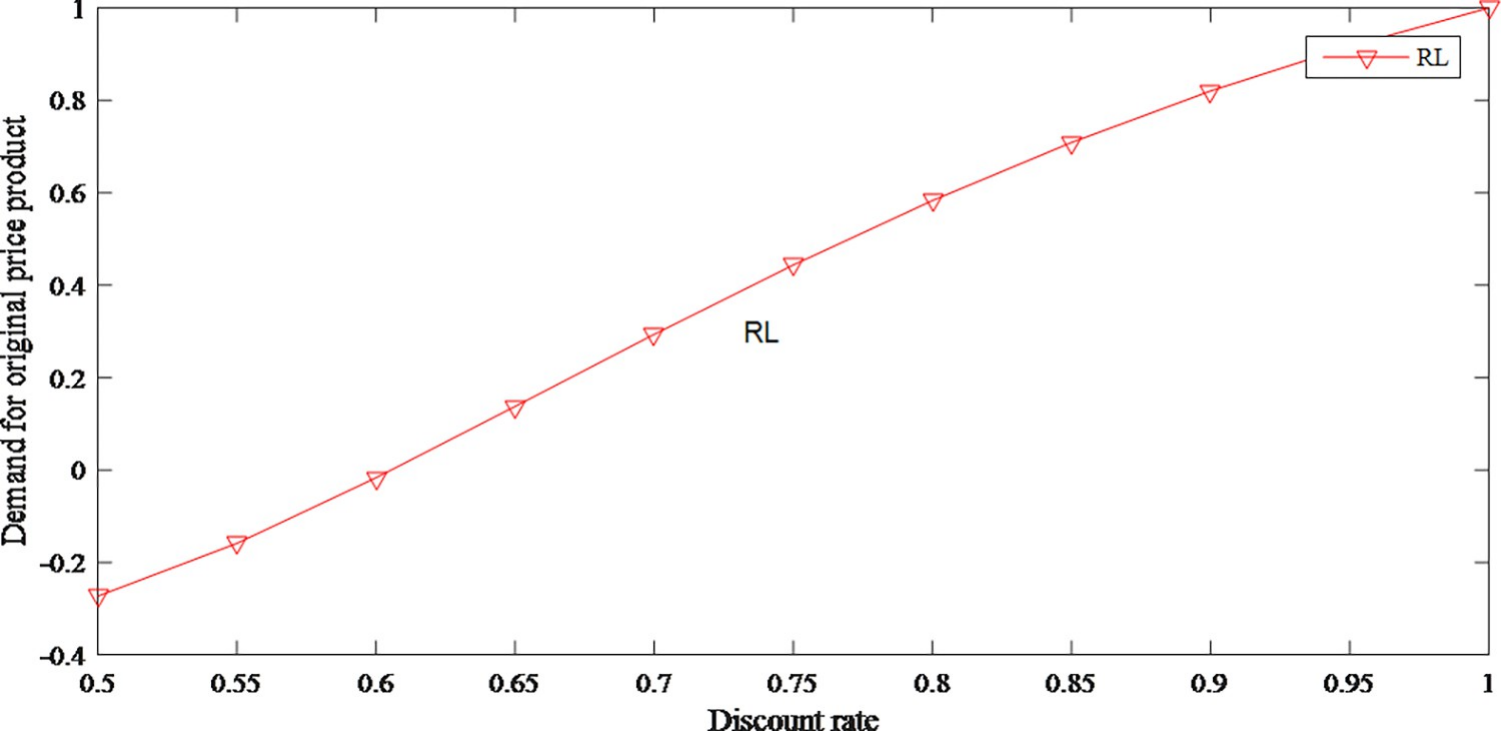
The impact of discount rate on demand for original price product.

**Fig 22:**
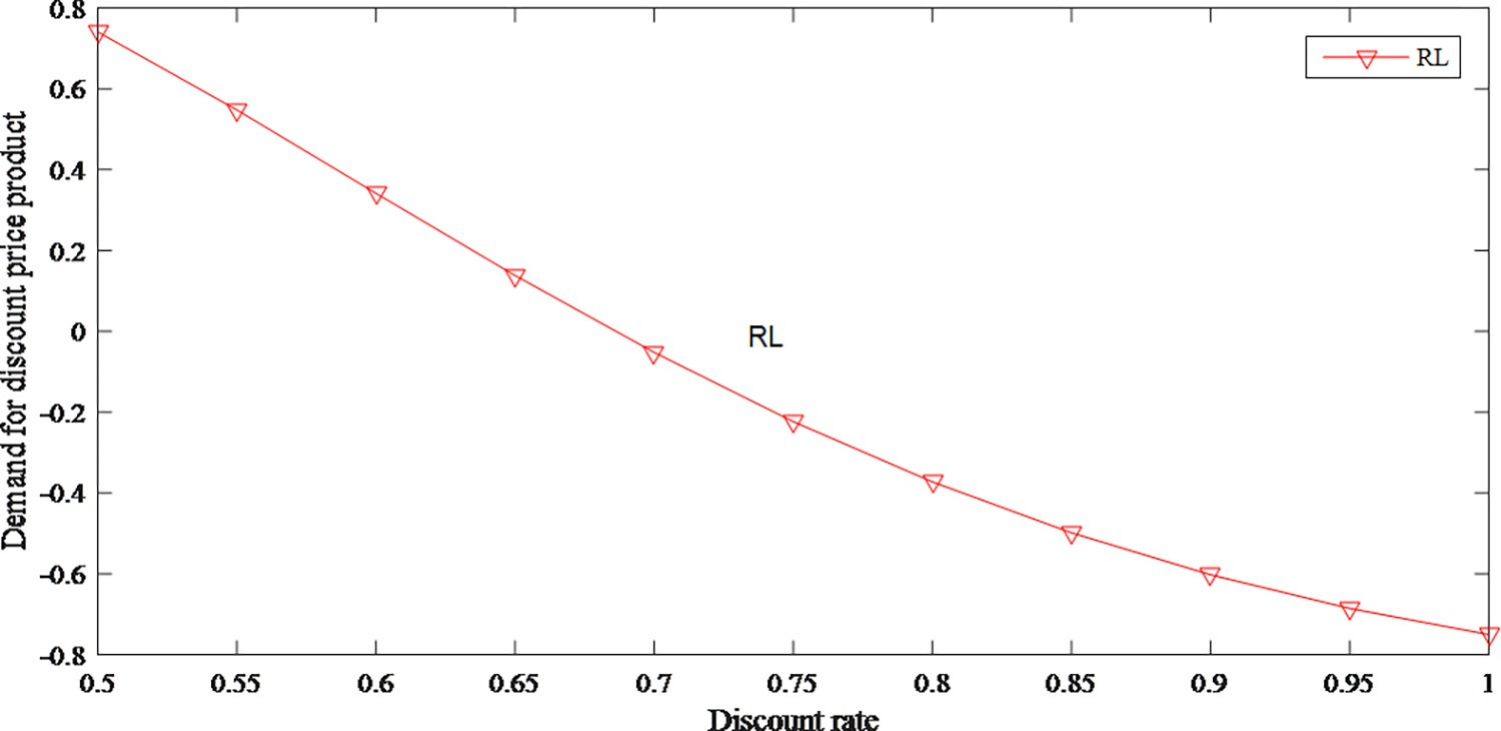
The impact of discount rate on demand for discount price product.

As shown in Figs 21 and 22, as the discount rate increases, original price product becomes more popular among consumers. When the sensitivity of regret is certain and the discount rate is low, consumers are more inclined to buy discounted products. When the sensitivity of regret is high, consumers would be more inclined to buy the original price product because they are afraid to regret it.

**Fig 23:**
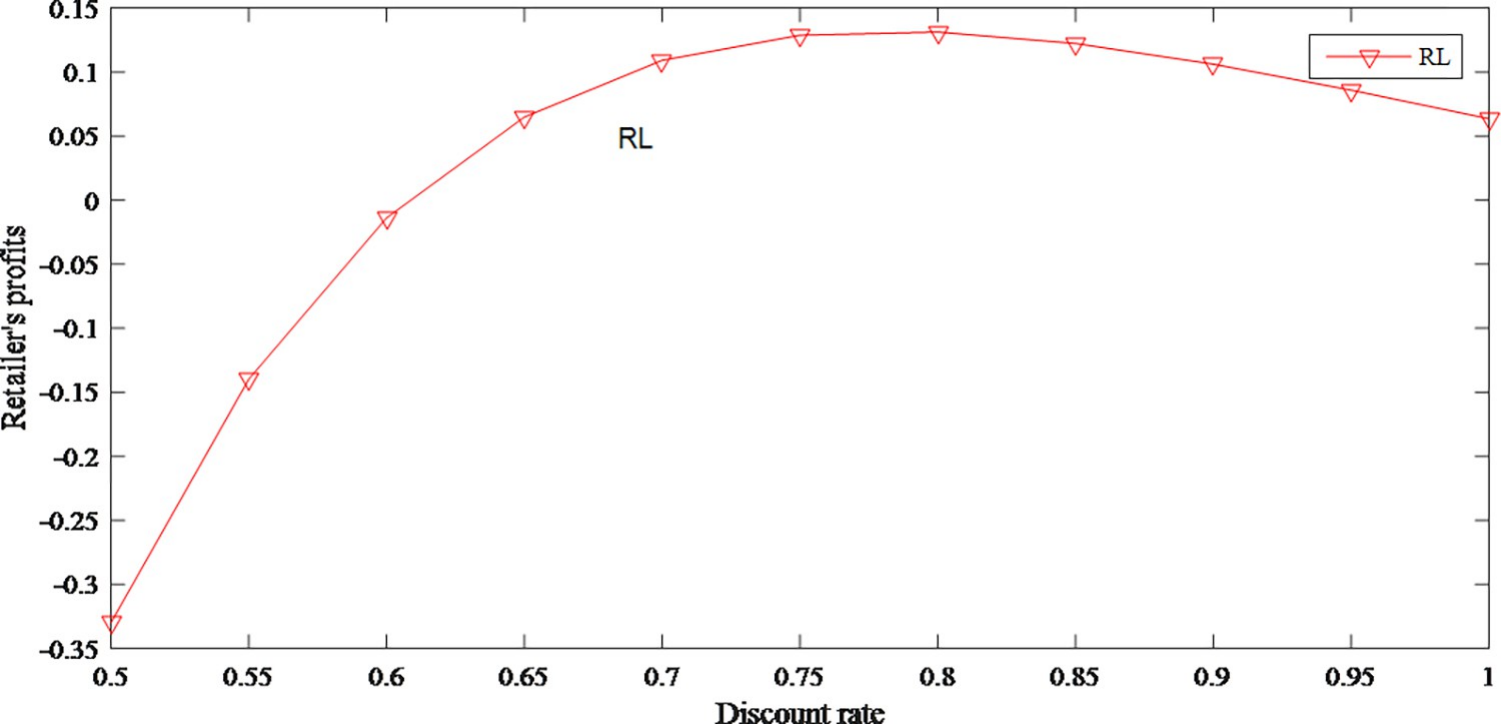
The impact of discount rate on retailer’s profits.

**Fig 24:**
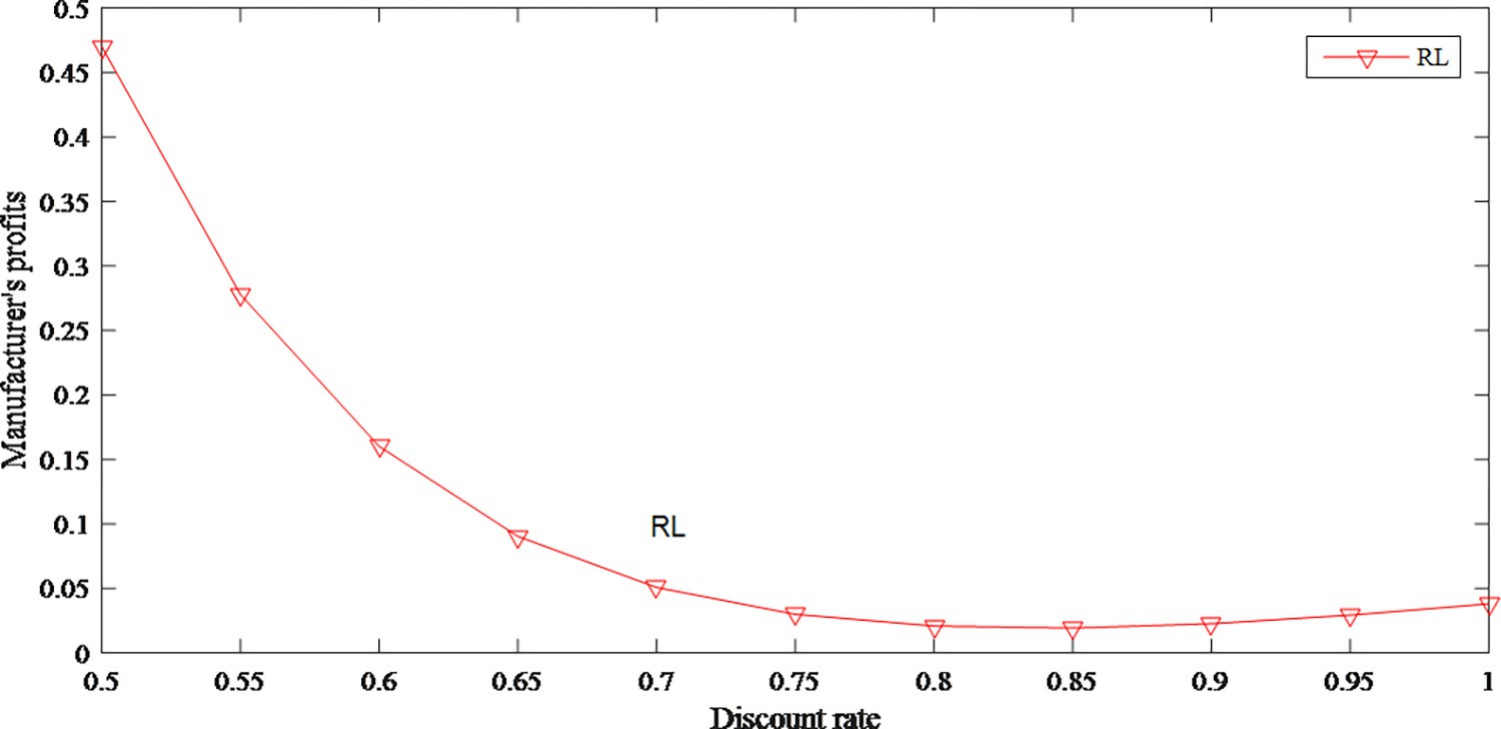
The impact of discount rate on manufacturer’s profits.

Due to the negative impact of the discount rate on product prices, to a certain extent, discount leads to a positive change in the demand for products. Then the retailer’s profits gradually increase. But the wholesale price decreases with the decrease of the retail price, so the manufacturer’s profits are gradually shrinking. However, retailer’s profits begin to shrink because the consumers’ demand declines under the effect of regret. At this time, wholesale price tends to stabilize, and manufacturer’s profits rebound slightly in the short term. The specific changes are shown in Figs 23 and 24.

### 5.5 A comparative analysis in model RL and model C

A comparative analysis is conducted to determine the difference between the model RL and model C.

**Fig 25:**
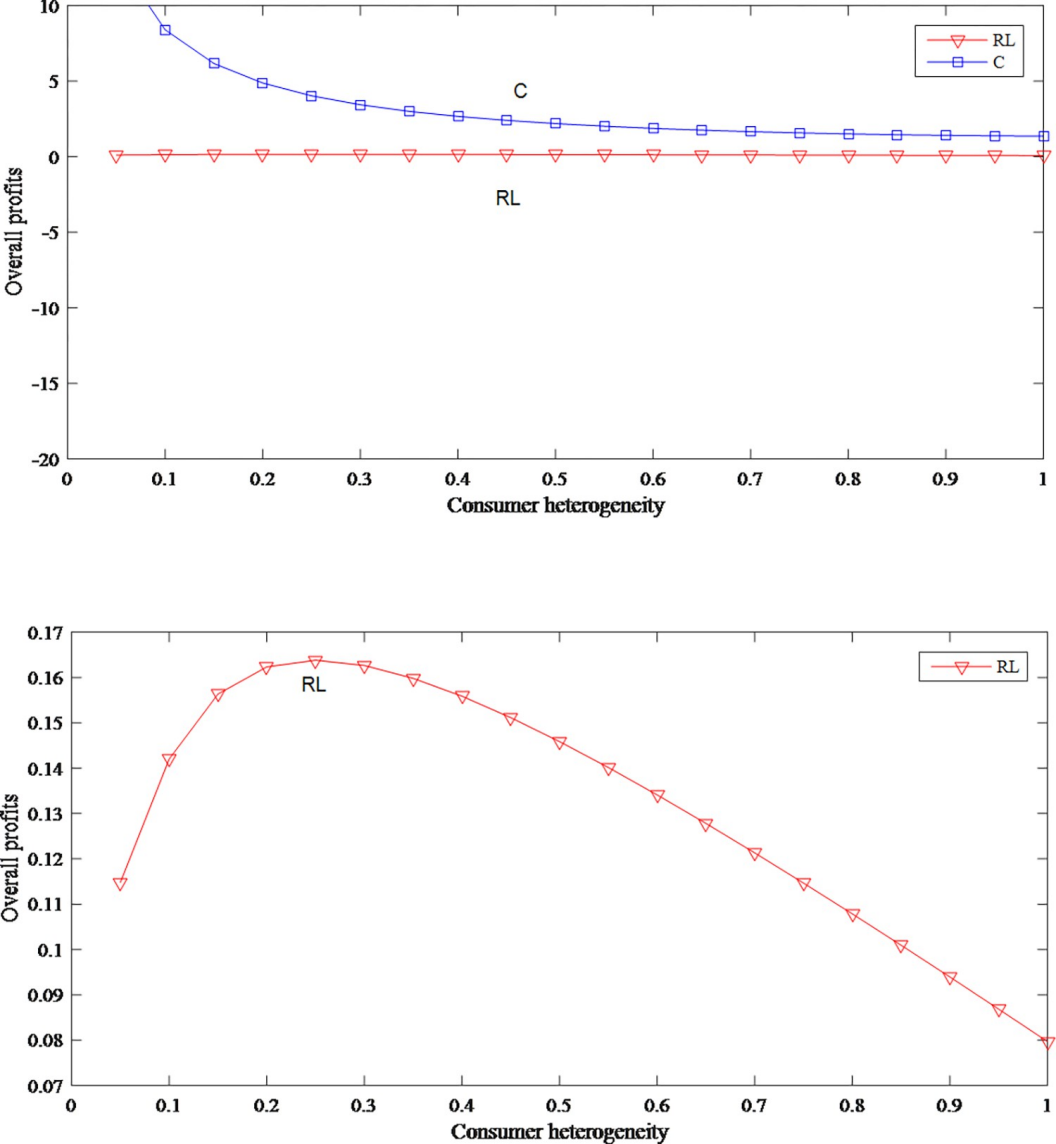
The impact of consumer heterogeneity on overall profits. (a) The overall profits in model RL and C, (b) The overall profits in model RL.

It can be seen from Figs 14 and 25 that whether it is in model C or in model RL, as the consumer heterogeneity increases, retail price and wholesale price are on a downward trend. In model RL, the overall profits first increase and then decrease, but compared with the profits in model RL, the retail price and the overall profits in model C are higher.

### 5.6 Analysis of the effect of revenue-sharing contract

To verify the validity of the revenue-sharing contract, assume *d* = 0.88, *γ* = 0.3, *c* = 10, *r* = 0.6, *f* = 0.2.The range of *φ* is [0.332451,0.998212] by calculation.

**Fig 26:**
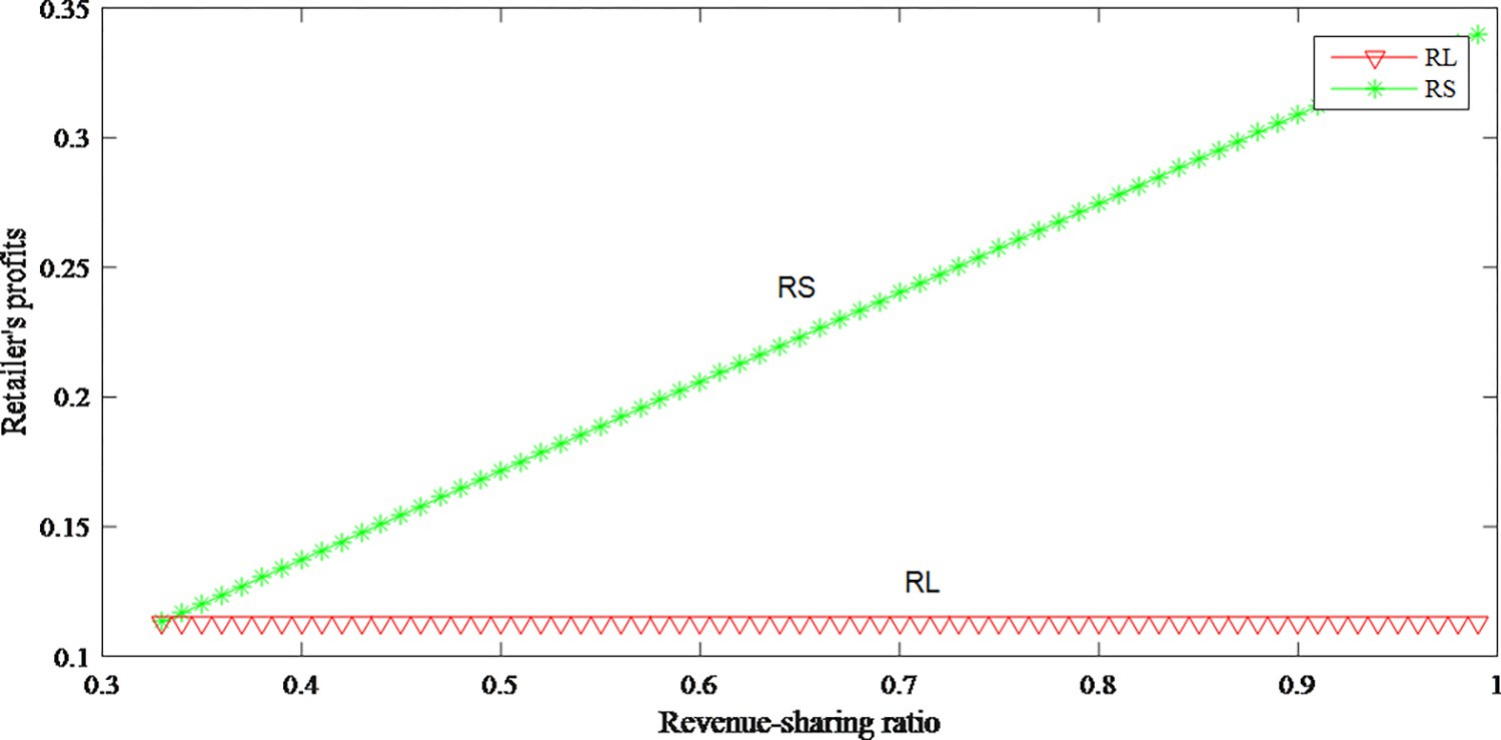
The impact of revenue-sharing contract on the retailer’s profits.

**Fig 27:**
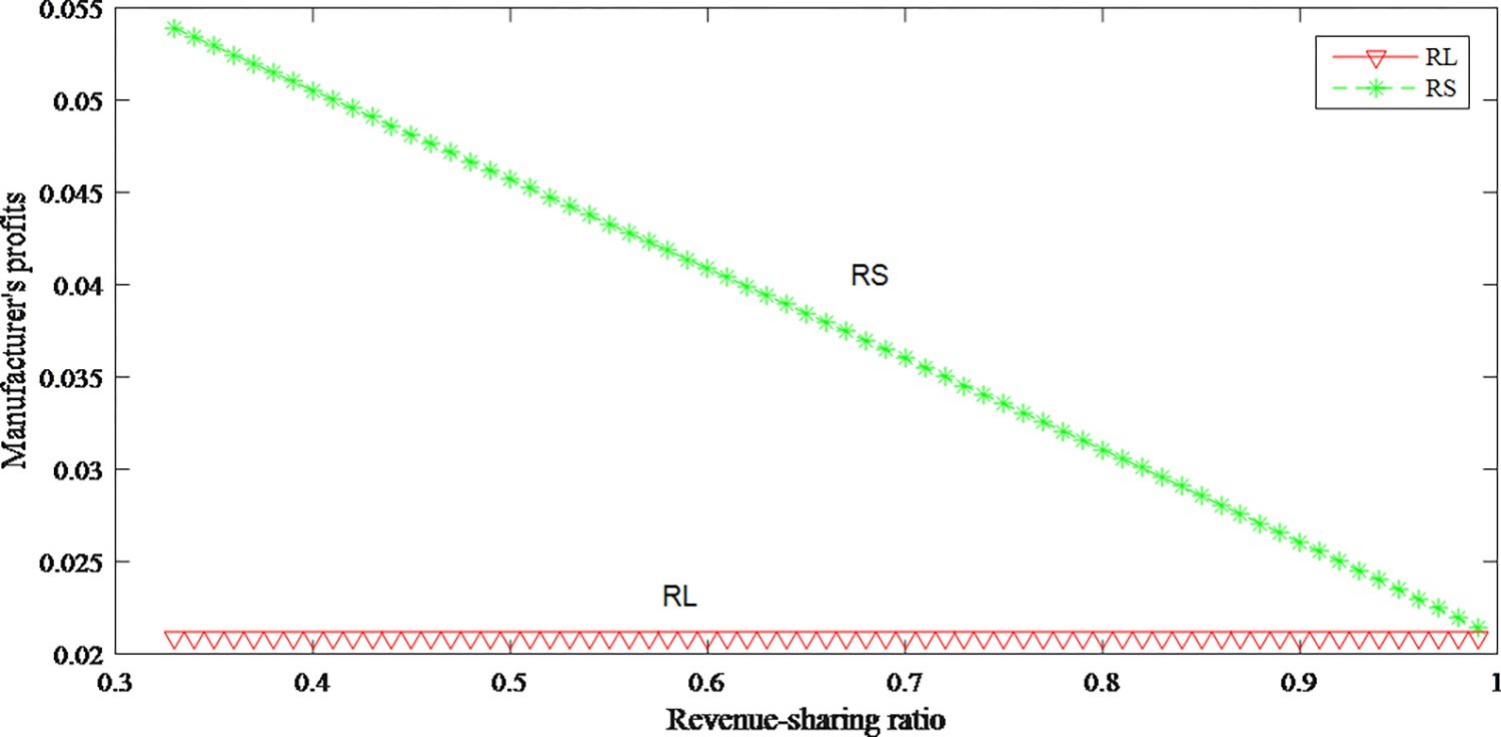
The impact of revenue-sharing contract on the manufacturer’s profits.

**Fig 28:**
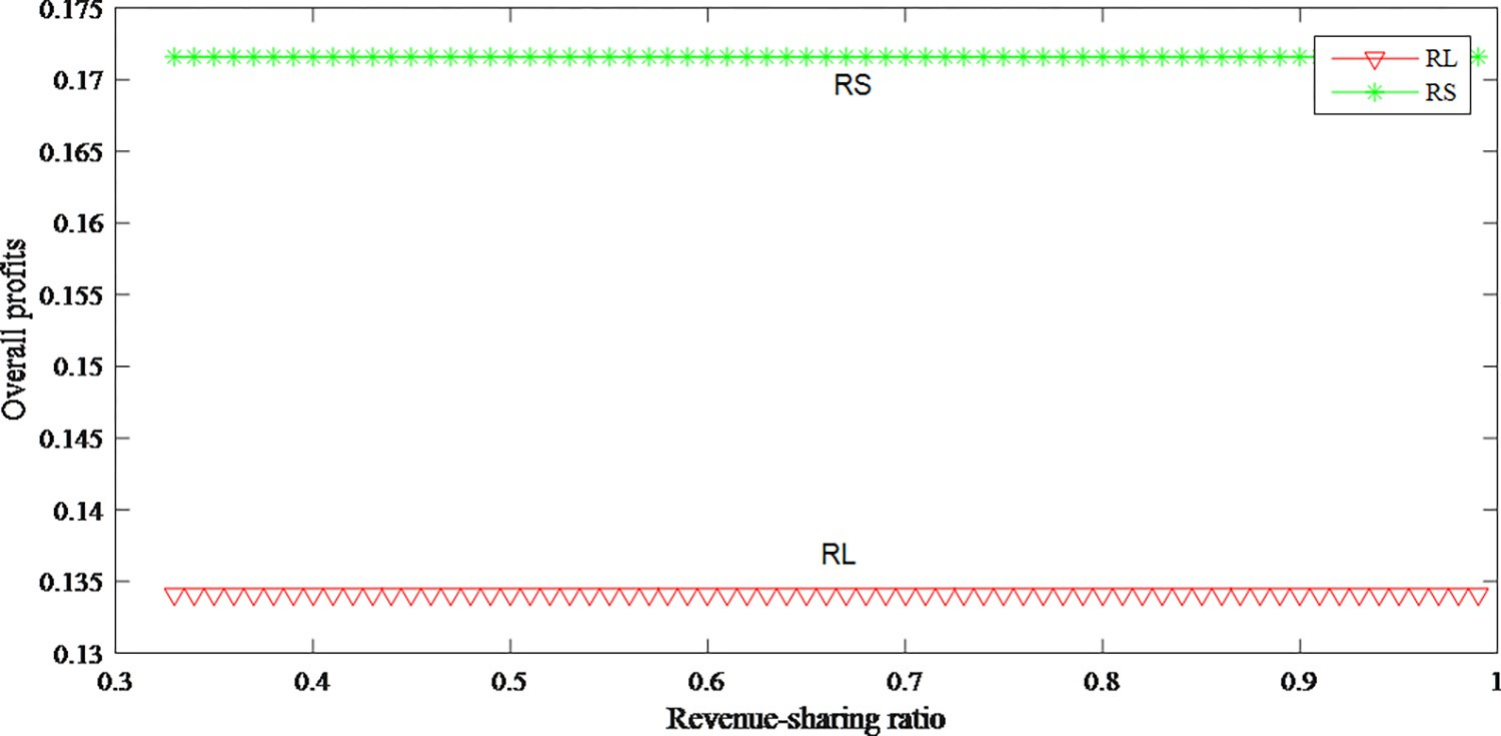
The impact of revenue-sharing contract on the overall profits.

Figs 26–28 reflect that the profits of both parties increase after revenue sharing. It can be seen from Fig 26 when retailer shares a larger proportion of revenue with manufacturer, retailer can obtain lower wholesale price, so that retailer can further reduce retail price and expand sales volumes to gain more profits. From Fig 27 that manufacturer’s profits decrease with the increasing sharing ratio. Although the manufacturer has obtained a larger proportion of revenue, the wholesale price has decreased, and the manufacturer’s unit production cost has not changed, so the decline in the unit profit ultimately leads to a loss. The overall profits after revenue-sharing have been greatly improved from Fig 28.

To sum up, in the retailer-led supply chain, retailer has the advantage of being close to the consumers and can get more profits. while manufacturer does not have leadership and does not know lots of information about market price and demand, a higher revenue-sharing ratio actually lowers profits. But in general, a revenue-sharing contract can increase the profits of all parties.

## 6. Conclusions

By constructing a retailer-led supply chain game model, pricing decision-making was discussed with considering the consumers’ anticipated regret and manufacturer’s fairness concerns. Comparison and the numerical simulation were adopted to analyze the influence of regret sensitivity, fairness concerns and price discount on the supply chain. Finally a revenue-sharing contract was designed to achieve the coordination. The several conclusions were as follows. (1) As anticipated regret rise, profits for both manufacturers and retailers will suffer. Companies have to lower the price to attract consumers, and due to the increasing degree of anticipated regret, consumers no longer favor the discounted products, which leads to increased demand for the original price products. Considering the consumer’s anticipated regret, the retailer should use the discount strategy carefully. (2) As fairness concerns increase, both retail and wholesale prices will increase, leading to lower demand and lower profits for retailer and manufacturer, which reminds retailers to pay attention to the uneven distribution of profits for manufacturers in the market. (3) Retail prices varied with price discount coefficients and consumer heterogeneity under the centralized decision-making model, which first increased and then decreased, but the retail price and wholesale price decreased under decentralized decision-making model. At this time. The price became a key factor that influenced the market demand, which was beneficial to the retailer but not to the manufacturer. (4) In the supply chain affected by consumers’ anticipated regret and manufacturer’s fairness concerns, a revenue-sharing contract can improve the efficiency of supply chain.

In this paper, we only consider the case of retailer dominance, and there are two market structures: manufacturer leadership and no leadership. And we did not consider the combination of different levels of regret expectations and fairness concerns. In the future there is still room for improvement. (1) The different power structures can be compared and analyzed. (2) the influence of different factors and their combination can be considered in the follow-up, such as consumers with high-level regret sensitivity and enterprises with low fairness concerns.
